# SOFI Simulation Tool: A Software Package for Simulating and Testing Super-Resolution Optical Fluctuation Imaging

**DOI:** 10.1371/journal.pone.0161602

**Published:** 2016-09-01

**Authors:** Arik Girsault, Tomas Lukes, Azat Sharipov, Stefan Geissbuehler, Marcel Leutenegger, Wim Vandenberg, Peter Dedecker, Johan Hofkens, Theo Lasser

**Affiliations:** 1 Laboratoire d’Optique Biomédicale, Ecole Polytechnique Fédérale de Lausanne, Lausanne, Switzerland; 2 Department of Chemistry, University of Leuven, Celestijnenlaan, Heverlee, Belgium; 3 Department of Radioelectronics, Faculty of Electrical Engineering, Czech Technical University in Prague, Prague, Czech Republic; University of California Berkeley, UNITED STATES

## Abstract

Super-resolution optical fluctuation imaging (SOFI) allows one to perform sub-diffraction fluorescence microscopy of living cells. By analyzing the acquired image sequence with an advanced correlation method, i.e. a high-order cross-cumulant analysis, super-resolution in all three spatial dimensions can be achieved. Here we introduce a software tool for a simple qualitative comparison of SOFI images under simulated conditions considering parameters of the microscope setup and essential properties of the biological sample. This tool incorporates SOFI and STORM algorithms, displays and describes the SOFI image processing steps in a tutorial-like fashion. Fast testing of various parameters simplifies the parameter optimization prior to experimental work. The performance of the simulation tool is demonstrated by comparing simulated results with experimentally acquired data.

## Introduction

The emergence of sub-diffraction fluorescence microscopy [1–5] has opened the door for novel insights in the life sciences by imaging features well beyond the diffraction limit [6]. Super-resolved single molecule localization methods such as photoactivation localization microscopy (PALM) [7] and stochastic optical reconstruction microscopy (STORM) [8] rely on stochastic emissions of photon bursts produced by independently blinking emitters. PALM and STORM analyze a sequence of image frames showing sparse sub-sets of emitting labels such that the emitters can be localized individually. The emitter localizations are then combined into a spatially super-resolved image of the sample.

In contrast to this frame-by-frame localization, super-resolution optical fluctuation imaging (SOFI) [9, 10] exploits the image sequence as a whole by using higher order statistics, i.e. higher order cross-cumulants to analyze the temporal fluctuations of blinking emitters for generating super-resolved images. The resolution enhancement increases with the growing cumulant order in all three spatial dimensions [11]. Balanced SOFI (bSOFI), an extension of SOFI, combines the information content of different cumulant orders further and allows one to extract physically meaningful parameters like density, brightness and blinking frequency of the observed blinking emitters [12].

Sample preparation for super-resolution imaging and an optimized choice of image acquisition parameters is often a tedious process requiring experience and several trials before a suitable parameter set is found. This work attempts to shorten this task by providing a simulation tool allowing a qualitative assessment of SOFI under various conditions and to assist the user to better understand the full chain of processing steps for SOFI. The simulator can be used for optimizing various experimental parameters such as blinking rate, labeling density, as well as system parameters of the microscope and camera prior the final imaging.

## The SOFI principle

SOFI applies high order nonlinear statistics to exploit the temporal blinking sequence of fluorescent emitters [9, 10]. More precisely, SOFI is based on calculating spatio-temporal cross-cumulants to obtain a 3D super-resolved, background-free and noise-reduced image using a conventional widefield microscope. As stated in the work initiated by J. Enderlein [9], the fluctuating emitters should fulfill the following conditions:

1. The markers should switch between at least two optically distinguishable states, e.g. a dark and a bright state.
2. Each emitter switches between the states repeatedly and independently in a stochastic manner.
3. The point-spread image of each emitter has to extend over several camera pixels.

The image intensity of a randomly blinking emitter is spatio-temporally correlated with itself but uncorrelated with neighboring signals. Images of stochastically blinking emitters are recorded such that the PSF is spread over several camera pixels. As a consequence, the intensities recorded by each camera pixel over which the PSF spreads are likewise spatio-temporally correlated.

**Fig 1:**
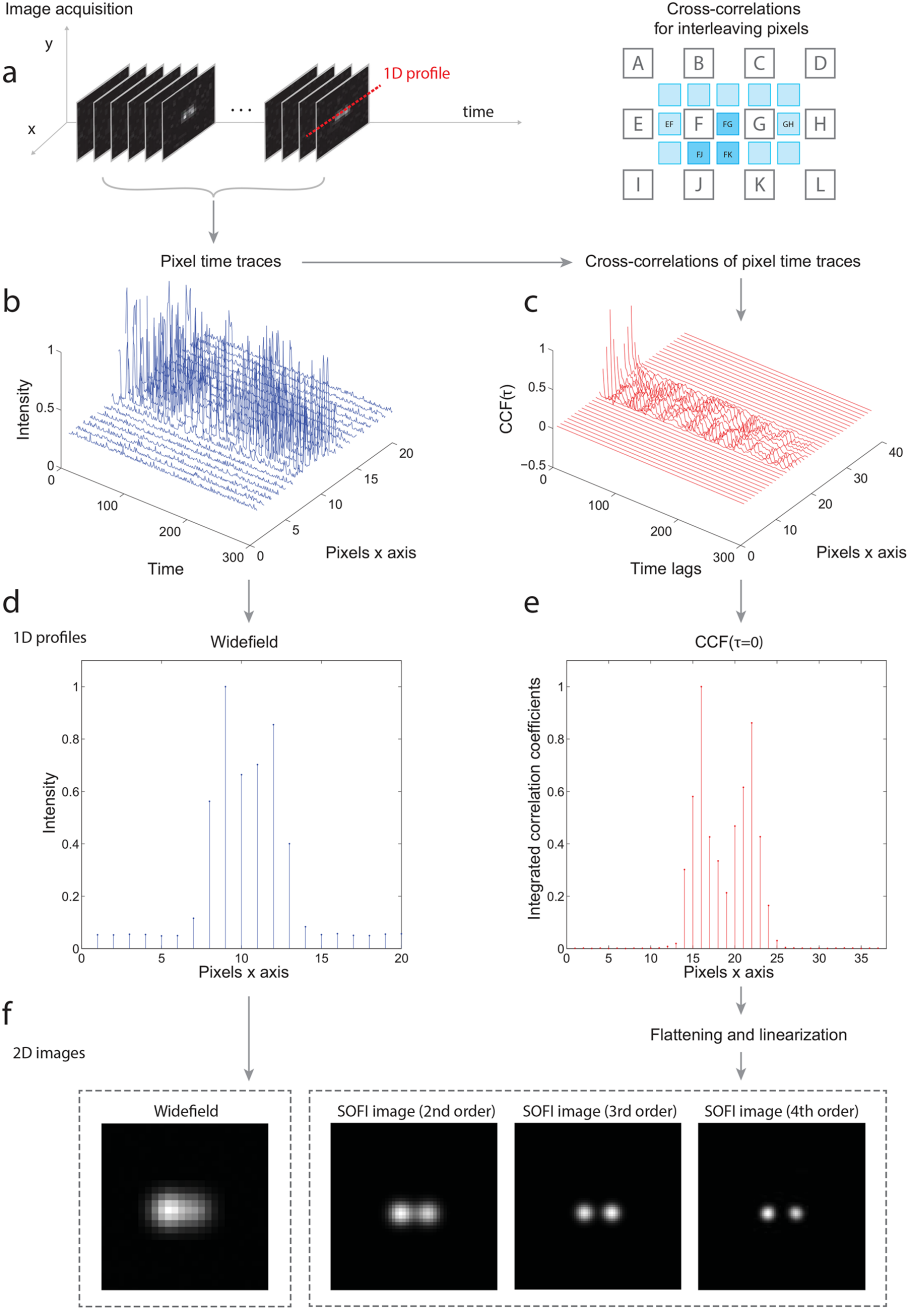
The SOFI principle in a one dimensional example. (a) 1D profile is taken from the input image sequence of two blinking emitters. (b) Corresponding 1D intensity time traces. (c) 2^*nd*^ order cross-cumulants calculated from the intensity time traces for all time lags. In practice, mainly the zero-time lag (*τ* = 0) is used. Using cross-cumulants, the interleaving pixels are also calculated. Note that the 2^*nd*^ order cross-cumulant is equivalent to cross-correlation. (d) The widefield image (the temporal average of intensity time traces). (e) The 2^*nd*^ order cross-cumulants for *τ* = 0. (f) The resulting 2D SOFI images up to the 4th cumulant order after flattening and linearization.

Fig 1 displays the general SOFI principle. By acquiring a stack of images, a time trace for each pixel is obtained. These pixel time traces contain all intensity contributions of each stochastically blinking emitter.

$$\begin{array}{c} I(r,t)=\sum_{k=1}^{M}\epsilon {}_{k}U(r-r_{k})s_{k}(t)+b(r) \end{array}$$ (1)

where *I*(**r**, *t*) denotes the intensity time trace at position **r**, *ϵ*_*k*_ the brightness, **r**_*k*_ the position and *s*_*k*_(*t*) normalized temporal fluctuations of the *k*^*th*^-emitter. *U*(**r**) is the PSF and *b* represents a stationary background. For each pixel, the *n*^*th*^ order cumulant is calculated for a better discrimination of emitters inside the PSF volume. Cumulants provide a correlative measure exhibiting the fundamental additivity property stating that the cumulant of the sum is the sum of cumulants, i.e. the cumulant analysis disentangles the emission patterns of closely spaced emitters [13]. By applying the *n*^*th*^ order cumulant to the Eq (1), we obtain

$$\begin{array}{ccc} \kappa_{n}\left\{I\left(r,t\right)\right\}\left(\tau \right) & = & \kappa_{n}\{\sum_{k=1}^{M}\epsilon_{k}U\left(r-r_{k}\right)s_{k}\left(t\right)+b\left(r\right)\}\left(\tau \right)\\ & = & \sum_{k=1}^{M}\kappa_{n}\left\{\epsilon_{k}U\left(r-r_{k}\right)s_{k}\left(t\right)\right\}\left(\tau \right)+\kappa_{n}\left\{b\left(r\right)\right\}\left(\tau \right)\\ & = & \sum_{k=1}^{M}\epsilon_{k}^{n}U^{n}\left(r-r_{k}\right)\kappa_{n}\left\{s_{k}\left(t\right)\right\}\left(\tau \right). \end{array}$$ (2)

For an *n*^*th*^ order cumulant, the PSF is raised to the *n*^*th*^ power. In consequence, the spatial resolution is improved by a factor of $\sqrt{n}$ [9]. Therefore, increasing the cumulant order yields an image with an enhanced spatial resolution. Since a multiplication in the spatial domain corresponds to a convolution in the spatial frequency domain, the cut-off frequency of *U*^*n*^(**r**) is in principle n-times higher than that of *U*(**r**). Consequently, by applying deconvolution and a subsequent rescaling, the cumulant image exhibits up to an n-fold resolution improvement [10].

An *n*^*th*^ order cumulant does not contain lower order correlation contributions which would hamper the resolution enhancement [9]. As an additional characteristic of cumulants, any non-fluctuating background is strongly suppressed. Additionally, SOFI processing, which relies on cross-cumulants, reduces uncorrelated noise [9, 10].

## Materials and Methods

### Sample preparation

Prior to transfection, HeLa [14] cells (American Type Culture Collection, ATCC CCL2) were incubated at 37°C with 5% CO_2_ using Minimum Essential Medium with Earles salts, L-glutamine, sodium bicarbonate complemented with 10% fetal bovine serum, 1× penicilin-streptomycin, 1× GlutaMAX, 1× MEM Non-Essential Amino Acids Solution (LifeTechnologies products). 4-well Nunc Lab-Tek II Chambered Coverglass (Thermo Fisher Scientific) was used as a chamber for the HeLa cells. Live HeLa cells were transfected with a pMD-Vim-Dreiklang plasmid using FuGENE 6 transfection reagent (Promega) and images were acquired at room temperature.

### Microscope setup

The microscope setup comprised a 60× water-immersion objective with a numerical aperture of 1.2 (UPLSAPO 60XW, Olympus), a green DPSS laser (MLL-FN-532, 800mW, Roithner Lasertechnik) for excitation and a 405nm diode laser (iBeam smart, 405 120mW, Toptica) for reactivation and tuning the blinking rate. 3D multiplane imaging was performed with a custom-built microscope setup presented in [11]. We used three 50:50 beam splitters (BS013, Thorlabs) and two sCMOS cameras (ORCA Flash 4.0, Hamamatsu). For 2D imaging, we used an additional 365nm illumination from a LED to tune the switching kinetics of the fluorescent protein Dreiklang [15] and an EMCCD camera (Andor iXon DU 897).

## Results

### The SOFI simulation tool

**Fig 2:**
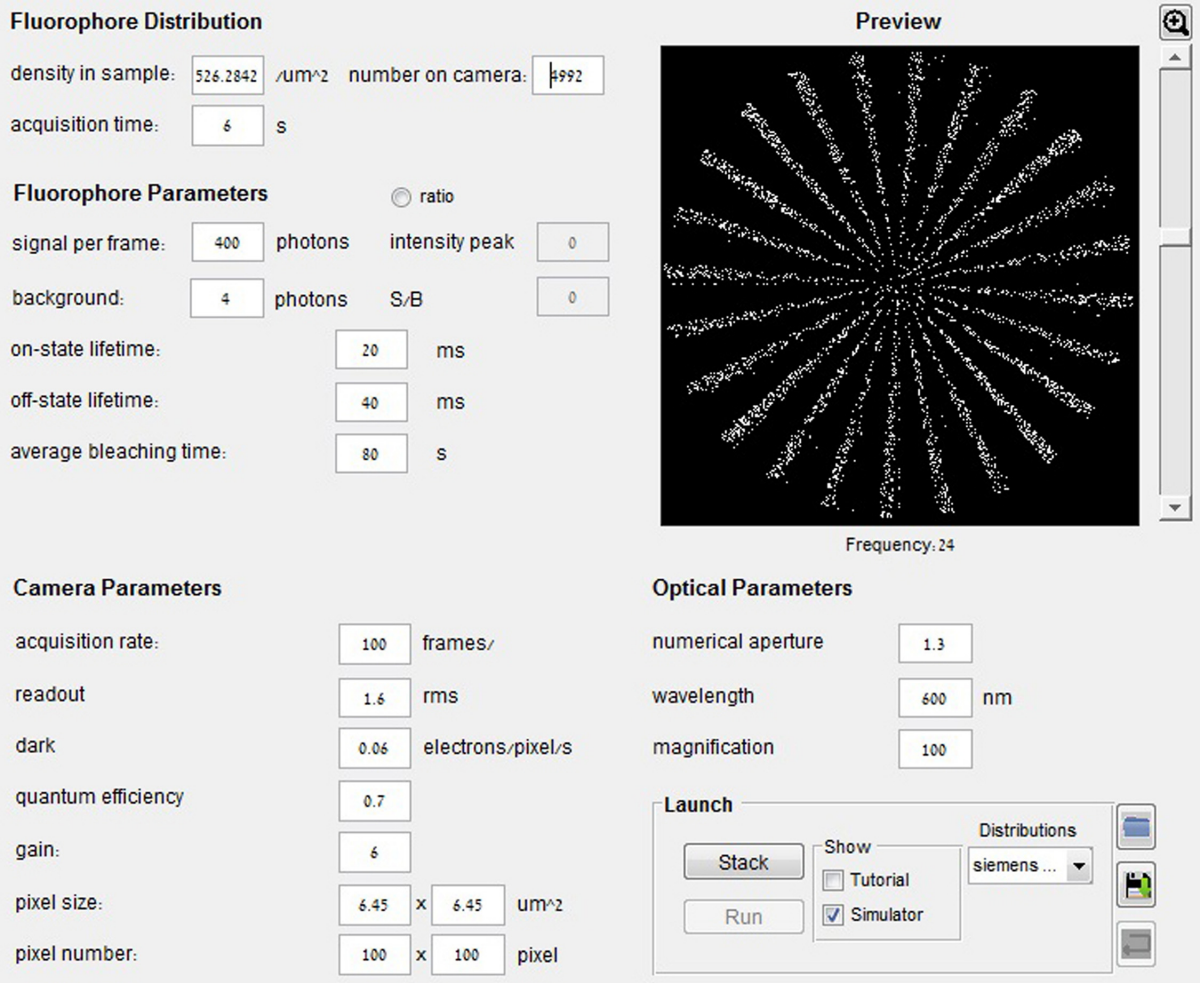
Screenshot of the main menu of the SOFI simulation tool. The user can specify the fluorophore distribution, various parameters of the fluorophores, camera and optics. For more details, see S1 Appendix.

We developed an simulation tool equipped with a graphical user interface (GUI). The microscope settings and the fluorescent sample can be investigated prior the experimental work. For more details about the GUI in Fig 2, please refer to the S1 Appendix. The software written in MATLAB is freely available together with a user manual at the website [16].

### Simulated data sets

The simulation starts by generating randomly placed emitters given by user-defined parameters such as the spatial density and spatial distributions. For each emitter, the blinking behaviour is modelled as a time-continuous Markovian process with an average blinking rate of $k=\frac{1}{\tau_{on}+\tau_{off}}$. For the experiments, we further describe the blinking rate by the on-time ratio defined as

$$\begin{array}{c} \rho_{on}=\frac{\tau_{on}}{\tau_{off}} \end{array}$$ (3)

The contribution *v*(*x*, *y*) of each fluorophore is given as [17]

$$\begin{array}{c} v(x,y\left|\hat{x},\hat{y},{\hat{\sigma }}_{xy}\right)=I_{x}I_{y} \end{array}$$ (4)

$$\begin{array}{ccc} I_{x} & = & \frac{1}{2}\text{erf}(\frac{x-\hat{x}+\frac{1}{2}}{\sqrt{2}{\hat{\sigma }}_{xy}})-\frac{1}{2}\text{erf}(\frac{x-\hat{x}-\frac{1}{2}}{\sqrt{2}{\hat{\sigma }}_{xy}})\\ I_{y} & = & \frac{1}{2}\text{erf}(\frac{y-\hat{y}+\frac{1}{2}}{\sqrt{2}{\hat{\sigma }}_{xy}})-\frac{1}{2}\text{erf}(\frac{y-\hat{y}-\frac{1}{2}}{\sqrt{2}{\hat{\sigma }}_{xy}}) \end{array}$$

where $\left(\hat{x},\hat{y}\right)$ describe the position related to the discrete pixel grid, i.e. within a circle of radius $3{\hat{\sigma }}_{xy}$. The PSF is assumed to be a symmetric 2D Gaussian with a standard deviation ${\hat{\sigma }}_{xy}$ determined by user-defined parameters (numerical aperture, camera pixel size and wavelength). The time varying brightness is generated for each blinking fluorophore. The signal per frame is obtained by summing contributions of all fluorophores at that time point. This procedure is performed frame by frame for simulating an acquired image stack. The simulation allows to add a background. Each pixel intensity is subjected to an additive Poissonian noise contribution. The light intensity per pixel is converted to an electric charge according to the quantum efficiency and gain of the camera setting. This electrical charge is modelled by a Gamma distribution Γ(*k*, *g*). Its shape *k* is given by the number of photons registered by each camera pixel and g is the camera gain. Finally, Gaussian noise with a standard deviation related to dark noise is added.

The program also includes a parameter which sets the characteristic time during which the fluorophore stays emissive before bleaching (the average bleaching time). The simulation models overall bleaching composed of switching fatigue and classical bleaching via excited states. Under constant illumination, as typically applied in SOFI, photo-switching and fluorescence excitation are proportional, such that our model describes the photo-bleaching satisfactorily well. The average bleaching time can be estimated from an experiment by an exponential fit to the plotted average fluorescence per frame. In contrast to the simulation, an initial transient can be present in a measurement until the fluorophores reached the dynamic switching equilibrium. The average fluorescence should then fade approximately exponentially towards an eventual background value.

In the simulation, all emitters exhibit the same on-state lifetime *τ*_*on*_ and off-state lifetime *τ*_*off*_. However in practice, the blinking statistics may spatially vary across the biological sample in a sample dependent yet uncontrollable manner.

### Implemented algorithms

**Fig 3:**
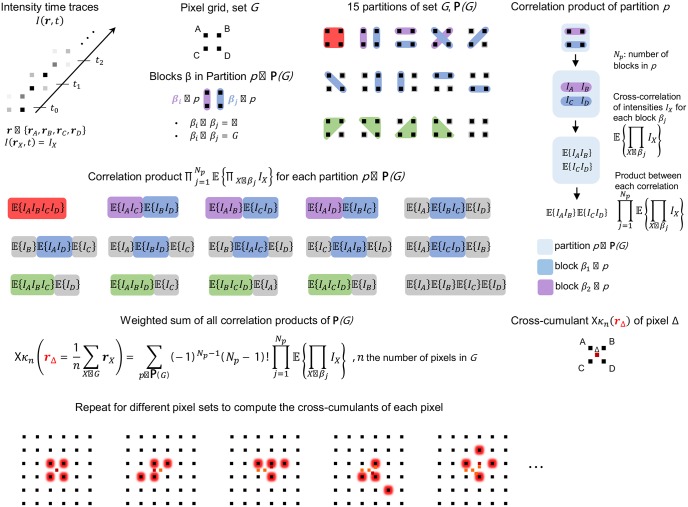
SOFI algorithm, cross-cumulant calculation. The *n*^*th*^ order cross-cumulant *κ*_*n*_ of a pixel *δ* is calculated as a weighted sum over all partitions of a set *G* of *n* pixels. The position of pixel *δ* is given by the geometrical mean of the *n* pixels within *G*. By using different sets of *n* pixels, the *n*^*th*^ order cross-cumulant of an arbitrary large pixel grid can be calculated. Formulas are shown for any *n* and sketches for *n* = 4.

The simulation tool incorporates SOFI and bSOFI algorithms [12]. Fig 3 shows the principle of the calculation of spatio-temporal cross-cumulants. By using spatial cross-cumulants, virtual pixels can be calculated in between the physical camera pixels leading to an image with a finer sampling grid [10]. A subsequent flattening operation corrects for differences in brightness between the physical pixels and virtual pixels of raw cumulants. The cumulant analysis leads to a nonlinear response to the brightness of fluorophores. The bSOFI algorithm introduces a linearization step for rescaling and linearizing the brightness response.

The simulation tool also incorporates a basic STORM algorithm [18] consisting of segmentation of each frame and subsequent localization of single molecules using Gaussian fitting methods. The image segments of a frame are generated by first applying a Laplacian of a Gaussian filter in order to reduce noise and to enhance the single emitter pattern (including a background subtraction). Once the image is segmented, a fitting procedure is performed on the raw data. An unweighted least-squares optimization based on the Levenberg-Marquardt algorithm estimates the amplitude, position, width, and background signal for each molecule. The initial position used to initialize Gaussian fit is determined by estimating the center of gravity of each image segment. Finally, estimates of width and amplitude deviating significantly from their expected values are discarded and a super-resolved STORM image is generated. The simulation tool also provides FALCON [19], an algorithm for STORM which combines a sparsity-promoting formulation with a Taylor approximation of the PSF for high-density imaging.

### Simulation examples

**Fig 4:**
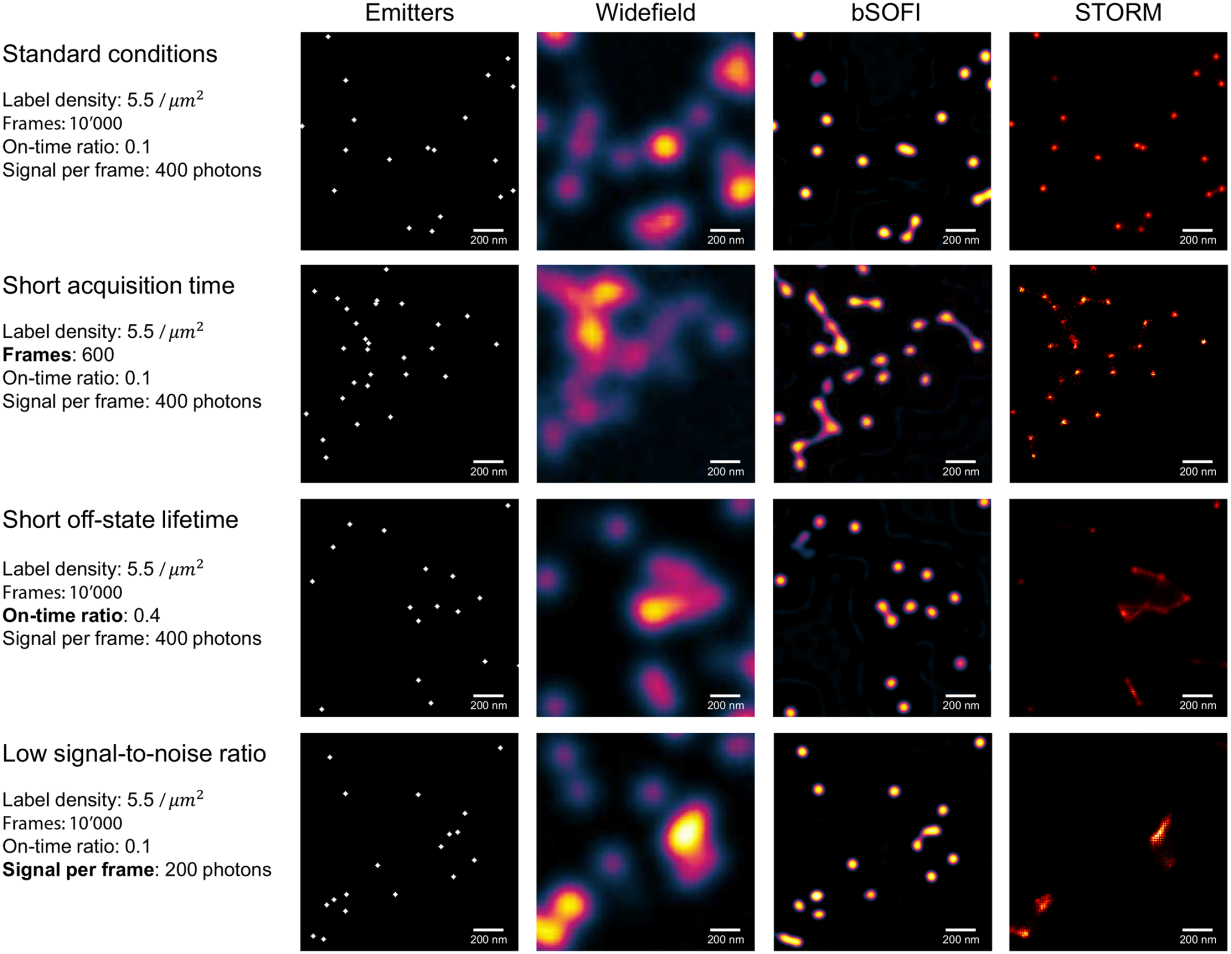
Simulation examples. Widefield, SOFI and FALCON STORM images with the generated emitter distributions for different imaging conditions. *Standard conditions* represent a scenario in which the simulator displays comparable performance for FALCON and SOFI in terms of resolution enhancement. All following experimental scenarios deviate from the standard conditions as follows: *Short off-state lifetime*, the sample is composed of emitters with fast off-switching kinetics; *Short acquisition time*, the super-resolution images are generated from an image sequence of only 600 frames; *low SNR*, the number of photons emitted per switching event per emitter is low which results in low signal-to-noise ratios (8 dB). Emitters shown in the left column are enlarged for the visualization purposes. All parameters of the standard conditions can be found in the S1 Appendix and on our project website [16].

We simulated various specific situations: standard conditions (i.e. conditions under which STORM is able to resolve individual emitters), short acquisition time, short off-state lifetime of the emitters, low signal-to-noise ratio (SNR), and high-labelling density. Reversibly photoswitchable fluorescent proteins have on-off duty cycles in the order of 0.1 in contrast to organic dyes with on-off duty cycles much smaller (≲ 0.01) [20]. Fig 4 shows the results for widefield, bSOFI, and FALCON STORM. For these simulations, we assumed fluorescent proteins and we set the on-time ratio to 0.1. Regarding the various conditions, bSOFI gave reliable results and seems to be well suited for photoswitchable proteins. The dark-state lifetime does not need to exceed the on-state lifetime and only needs to be on the order of the frame exposure time, which is in agreement with previous findings [18].

**Fig 5:**
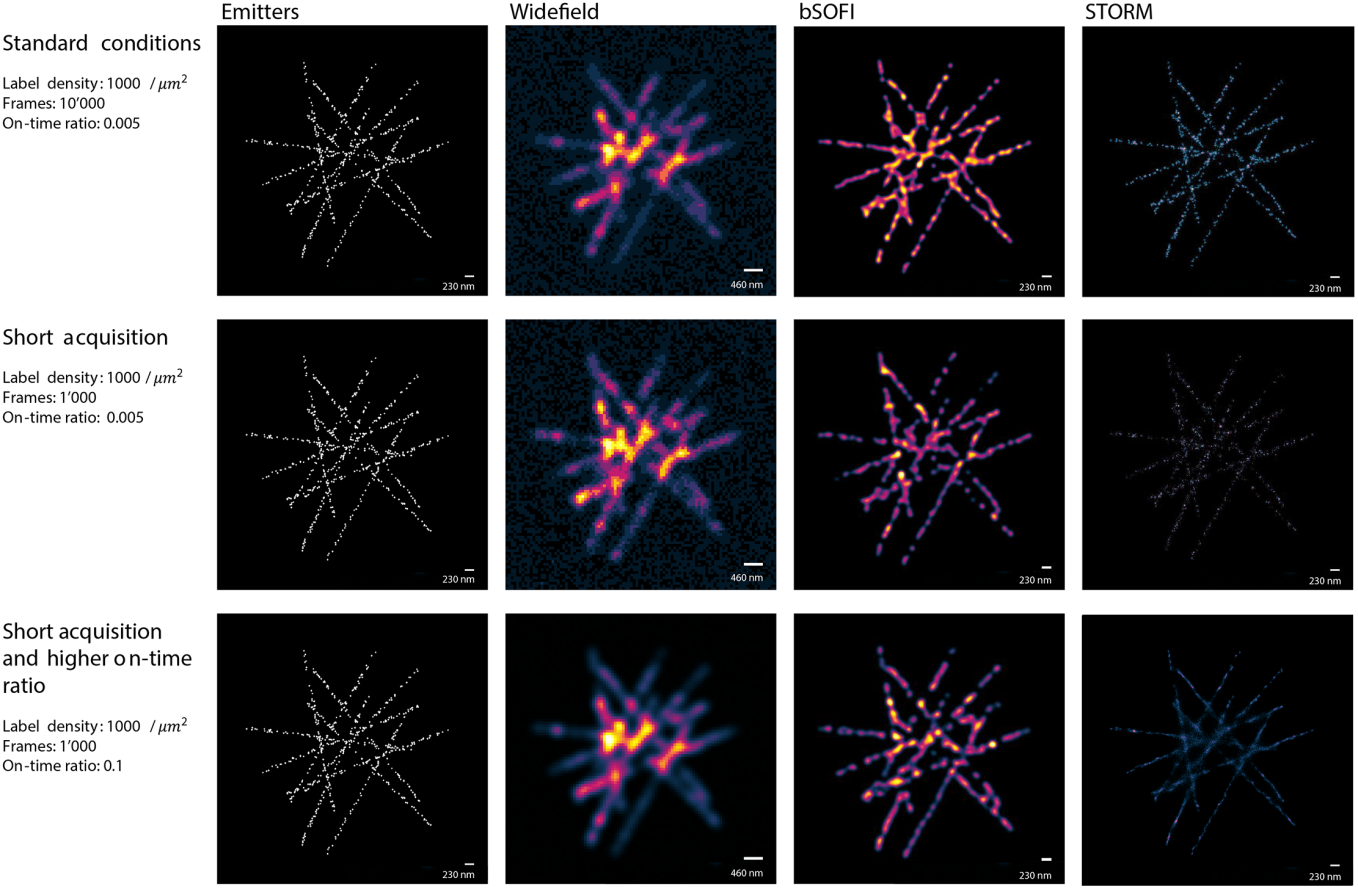
High-labelling density simulations. Widefield, SOFI and FALCON STORM images of the simulated structures labelled with a relatively high density of emitters (1000/*μm*^2^) *Standard conditions* represent a scenario well suited for STORM. in the case of *Short acquisition time*, the super-resolution images are generated from an image sequence of only 1000 frames. *Short acquisition time and higher on-time ratio* represent a situation with very high density of activated emitters per frame which makes it challenging for STORM algorithms. Emitters shown in the left column are enlarged for the visualization purposes. All parameters of the standard conditions can be found in the S1 Appendix and on our project website [16].

Fig 5 shows simulated structures such as filaments labelled with a relatively high density of emitters of 1000/*μm*^2^. In the case of a sufficiently long off-state life time of the emitters (on-time ratio ≲ 0.01), strong signals, and high number of frames (≈10000), STORM provides a high resolution enhancement, whereas SOFI can be useful even if these conditions are not met. The simulations reveal a good performance for bSOFI even in the case of a short off state lifetime of the blinking molecules and a relatively short acquisition time.

**Fig 6:**
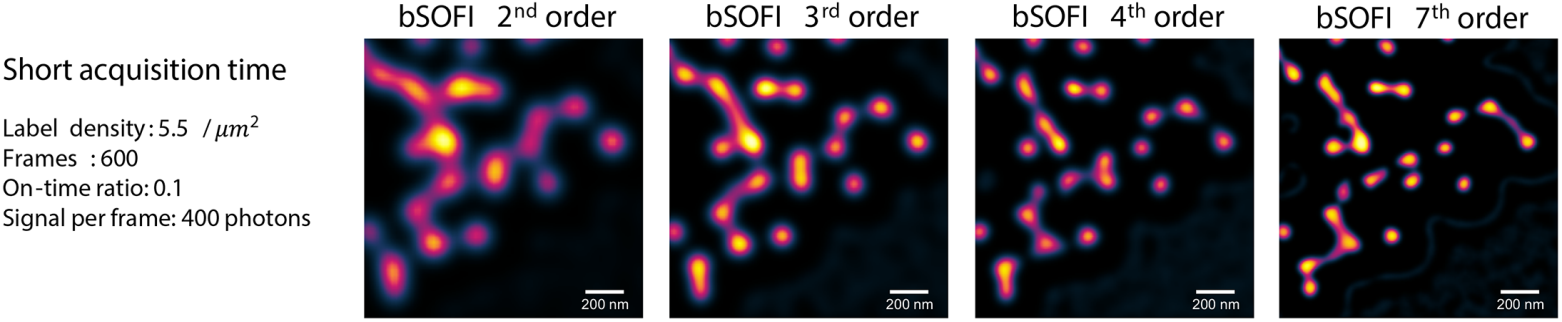
Balanced SOFI (bSOFI) images of different orders for the test case with only 600 input frames. With increasing order of the SOFI analysis, resolution improvement also increases, but higher orders generally require more input frames in order to avoid apparent artifacts.

The deconvolution step in bSOFI helps to exploit the maximum potential resolution improvement given by the order of the cumulant analysis, but it comes at the price of introducing common deconvolution artifacts. Deconvolution may cause ringing artifacts due to conversion of a discontinuous signal into Fourier space. These ringing artifacts appear mostly along sharp edges or points. For sufficiently high signals (SNR approx. 20dB), the artifacts have very low values and can be neglected. With increasing order of the SOFI analysis, resolution improvement also increases, but the SNR of super-resolved images decreases. This effect limits the highest resolution achievable in practice. The short acquisition case represents unfavorable conditions for the 7th order bSOFI shown in Fig 4. In that case, it is better to use a lower order SOFI analysis. Fig 6 shows bSOFI images of different orders. For lower order bSOFI images, the ringing artifacts are less pronounced and the two points in the top right corner are properly separated. Both STORM and SOFI have a range of optimal conditions. Our simulation tool can be used to quickly check various conditions and the effect on the output super-resolved images.

### Experimentally acquired images of live HeLa cells compared to simulations

**Fig 7:**
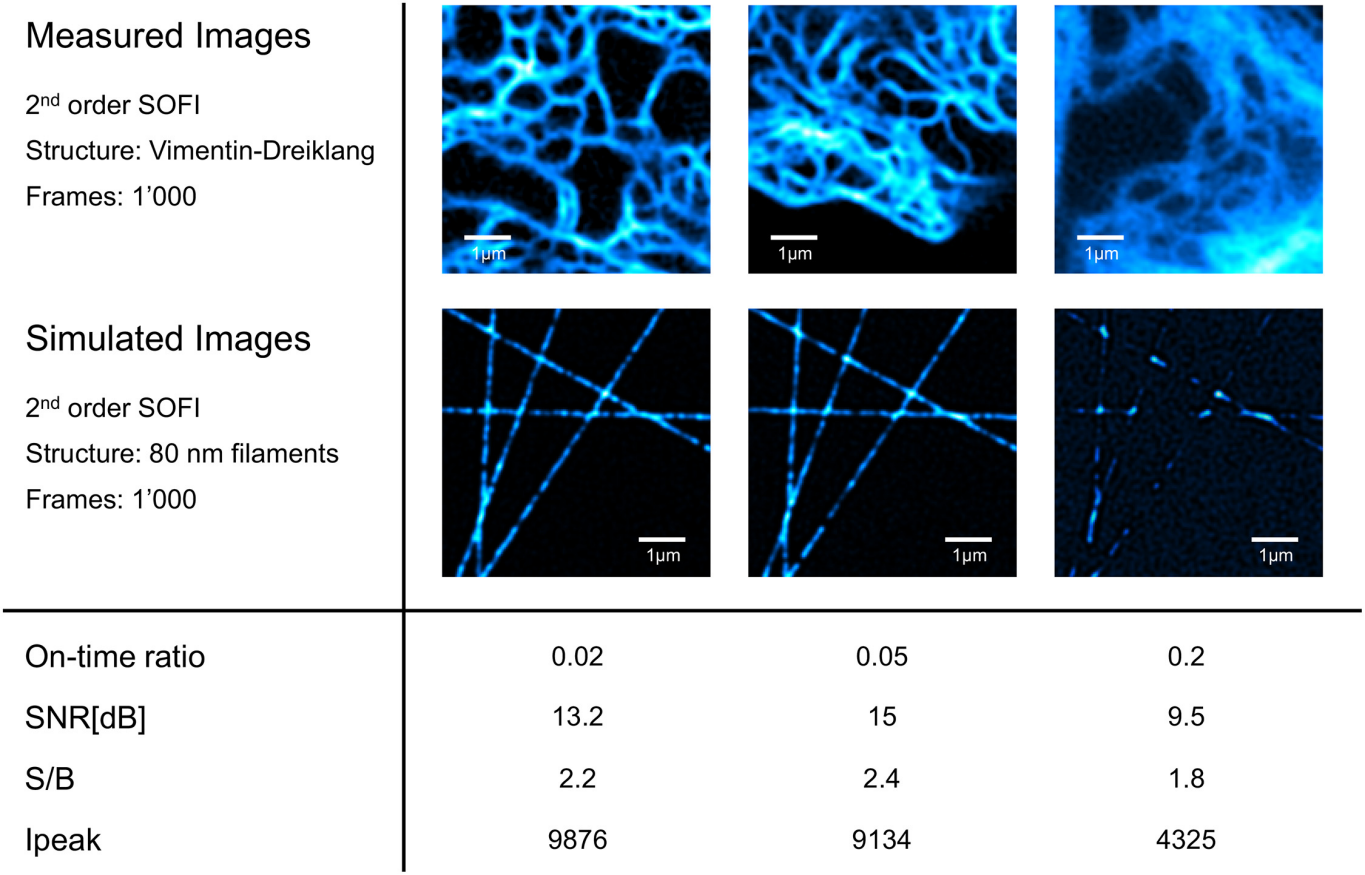
Experimental data compared to simulations. (a)-(d) 2^*nd*^ order bSOFI images computed from experimental data. (e)-(h) 2^*nd*^ order bSOFI images computed from simulated data. Table below the figure shows the parameters estimated from experimental data and used for the simulations. S/B and Ipeak denote respectively the signal-to-background ratio and the intensity peak of the average images. Density for simulation was set to 600 fluorescent proteins per micrometer.

We performed an experiment comparing simulated data to experimentally acquired data. Parameters of the experimentally acquired images were measured and used to set the simulation parameters in the GUI. The measured parameters (shown in Fig 7) were the peak signal (*I*_*peak*_), the peak signal to background ratio (*S*/*B*), molecular on-time ratio Eq (3) of Dreiklang fluorescent protein, and molecular density.

Fig 7a, 7b and 7c shows experimentally acquired images of Dreiklang-labelled vimentin networks in HeLa cells. By decreasing the 405nm illumination intensity, and increasing the 365nm intensity at the same time, the on-time ratios of Dreiklang can be tuned. The estimated on-time ratios in the acquired image sequences were 0.02, 0.05, and 0.2 accordingly. On-time ratios of Dreiklang were measured in live cells using a procedure described in [11]. An average image from each acquired image stack was calculated and thresholded by an iterative threshold selection method [21] in order to generate signal and background masks. The estimated SNRs and signal-to-background ratios (S/Bs) were measured from the average images according to the procedure described in [22] and used as simulation parameters. Under normal conditions in cells, we assume a few hundreds to thousand Dreiklang per micrometer of fiber length. For this simulation example, we set the density to 600 fluorescent proteins per micrometer. Number of frames of the simulated image sequence was set to 1000. Fig 7a, 7b, 7d and 7e shows two situations which lead to high quality images, and Fig 7c and 7f shows a situation which resulted in low quality images.

## Conclusions

We developed a novel software for modeling the imaging procedure of super-resolution optical fluctuation imaging. The software is equipped with a user friendly graphical interface which allows the user to generate simulated image stacks and calculate SOFI and STORM super-resolution images. The processing steps of SOFI are visualized and explained in a tutorial-like way. We compared simulated results with experimentally acquired data of living HeLa cells expressing vimentin-Dreiklang. We demonstrated that the software is able to predict, through simulation, the image quality. The software allows the user to quickly test numerous image acquisition settings prior to experimental work. For more information about the software, see S1 Appendix.

## Supporting Information

S1 Appendix User Manual. Documentation for installing and using the software.
(PDF)

S2 Appendix Software package. Zip file which includes the software package. The software is written in MATLAB, equipped with graphical user interface and freely available together with a user manual also at [16].
(ZIP)
