# Supplementary material for: SOFI Simulation Tool: A Software Package for Simulating and Testing Super-Resolution Optical Fluctuation Imaging
Source: PLoS One. 2016 Sep 1;11(9):e0161602. doi: 10.1371/journal.pone.0161602 (PMC5008722; doi:10.1371/journal.pone.0161602)
Supplement: S1 Appendix — Documentation for installing and using the software. (PDF) [file pone.0161602.s001.pdf]

User Guide

SOFI simulation tool: a software package for simulating and testing super-resolution optical fluctuation imaging

Arik Girsault[1], Tomas Lukes[1,3], Azat Sharipov[1], Stefan Geissbuehler[1], Marcel Leutenegger[1+], Wim Vandenberg[2], Peter Dedecker[2], Johan Hofkens[2], and Theo Lasser[1,*]

[1]Laboratoire d'Optique Biomédicale, Ecole Polytechnique Fédérale de Lausanne, Station 17, CH-1015 Lausanne, Switzerland
[2]Department of Chemistry, University of Leuven, Celestijnenlaan 200F, 3001 Heverlee, Belgium
[3]Department of Radioelectronics, Faculty of Electrical Engineering, Czech Technical University in Prague, Technická 2, 166 27 Prague 6, Czech Republic

[*] *tomas.lukes@epfl.ch,theo.lasser@epfl.ch*

Contents

1 About the User Guide **ii**

2 Graphical User Interface **1**
2.1 Start menu 2
2.2 Tutorial menu 6
2.3 Simulator menu 8

3 Appendix **9**

1 About the User Guide

The user manual provides a detailed description of the architecture and work flow of the *SOFI optimization tool*, a Matlab-based graphical user interface (GUI). The GUI is distributed under the terms of the GNU GPLv3 license and the source code is available at the project website: *http://lob.epfl.ch/sofitool.html*. The software has been tested with 32 and 64bit Matlab version 2014b and 2015a and requires the "Image Processing Toolbox". Please refer to the README.txt for launching the GUI. In case you encounter an error, please contact arik.girsault@gmail.com and describe the procedure to reproduce the error. Please specify also your Matlab version as well as your operating system.

2 Graphical User Interface

The flowchart of the SOFI simulator is shown in Figure 1. The program is divided into three parts: firstly the *start menu* in which the user enters the experimental parameters to generate image datasets; secondly, the *tutorial* displaying the recorded image sequence of blinking emitters and the SOFI processing steps in a one and two-dimensional 2nd order SOFI image sequence (more details see Fig. 3); and finally the simulator showing the distribution of fluorophores, the expected widefield, STORM as well as the SOFI images for each cumulant-order (Fig. 5).

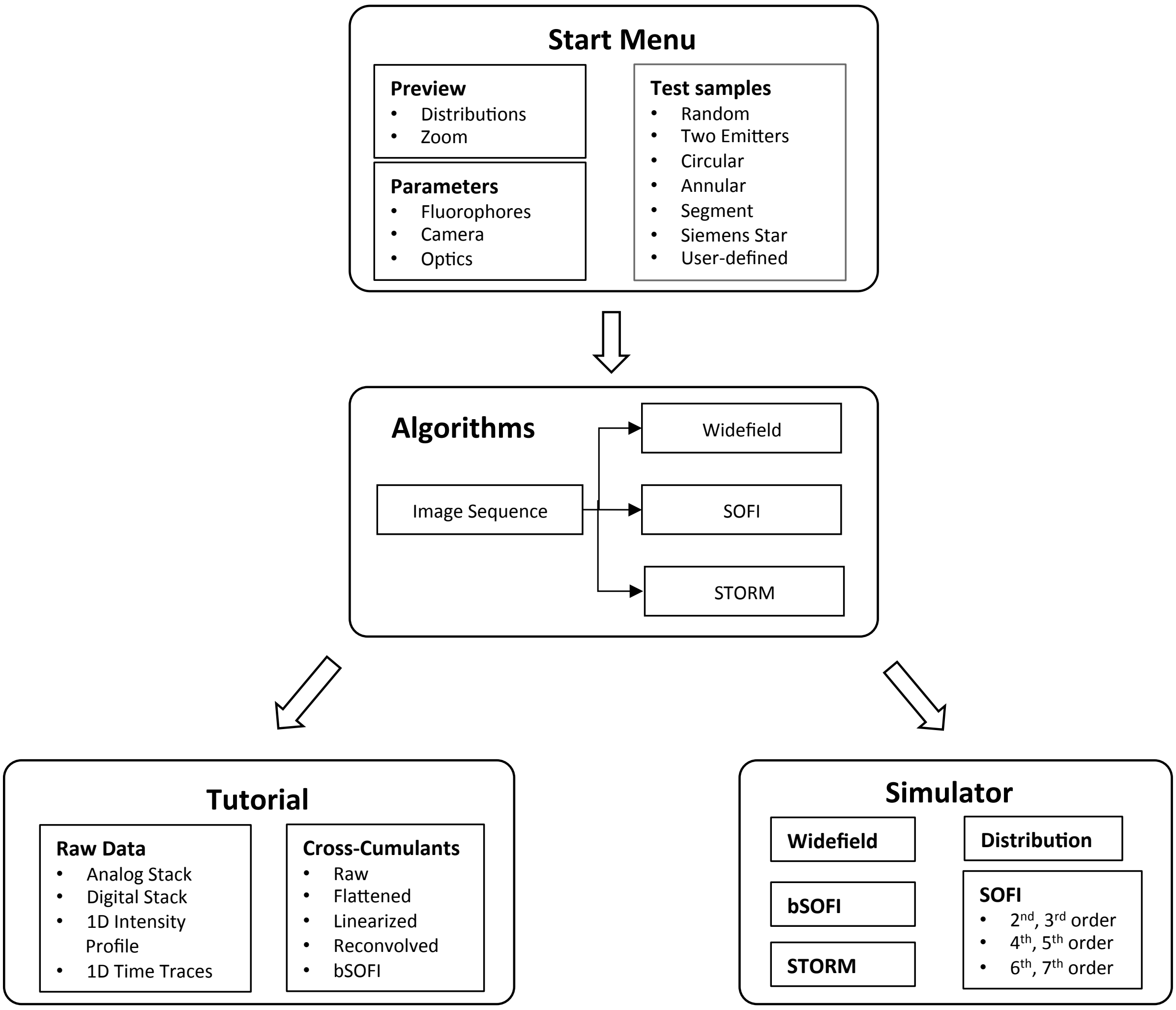

Figure 1: Flowchart showing the start menu, the software essential algorithms, the tutorial and simulator interfaces.

2.1 Start menu

The *start menu* asks the simulation parameters and is composed of three parts: a 2D image showing the spatial distribution of fluorophores, a parameter list subdivided into fluorophore, camera and imaging optics, and a panel to launch the simulation tool and the analysis.

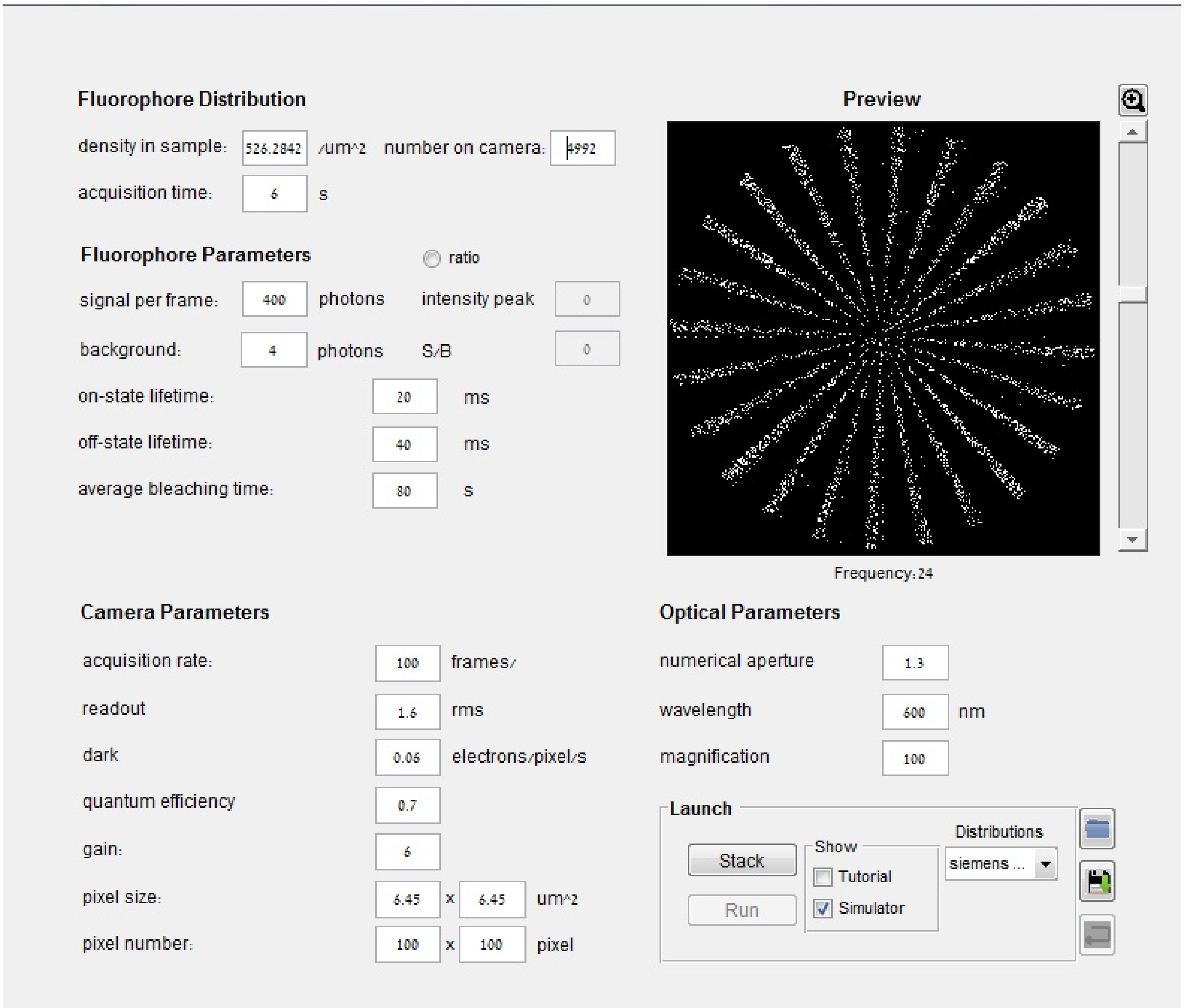

Figure 2: Start menu specifying the parameters of the simulation

The simulation parameters the user can specify are listed below along with a short description:

Fluorophore Distribution

- density in sample: density of fluorophores in the cell sample, units [$number\ of\ fluorophores/\mu m^2$]. This parameter is related to the number of fluorophores in the sample through the following equation:

$$density = \frac{number\ of\ fluorophores}{number\ of\ pixels\ in\ structure \times \left(\frac{camera\ pixel\ size}{optical\ magnification}\right)^2}$$

- number on camera: number of fluorophores in the field of view of the camera, units [$number\ of\ fluorophores$].

- acquisition time: time over which the camera records the fluorescence signal, units [s]. This parameter is related to the number of frames of the image sequence SOFI uses for computing cumulants through the following equation:

$$number\ of\ frames = acquisition\ time \times rate\ of\ acquisition$$

  with the rate of acquisition defined below in the section *Camera Parameters*.

Fluorophore Parameters

- signal per frame: number of photons emitted by a single fluorophore in a frame, units [$number\ of\ photons$]. Alternatively, by selecting the *ratio button*, the user can specify the intensity peak of the target widefield microscopy image, units [$grayscale$].

- on-state lifetime: average time during which the fluorophore is active, i.e. emitting photons, units [ms].

- off-state lifetime: average time during which the fluorophore is inactive, i.e. dark, units [ms].

- average bleaching time: average time it takes the fluorophore to bleach, i.e. remain in an irreversible dark state, units [s].

- background fluorescence: fluorescence not emitted from the target molecule, units [$photons$]. In a cell sample, this background could arise from auto-fluorescence, i.e. fluorescence emission from small biological molecules such as NADH. Alternatively, by selecting the *ratio button*, the user can specify the signal-to-background ratio of the target widefield microscopy image, units [$grayscale$].

Camera Parameters

- acquisition speed: number of frames acquired by the camera per second, units [$frames/s$].

- readout noise: combination of the noise introduced by the system converting photons to electrons, the subsequent processing and analog-to-digital conversion, units [$root\ mean\ square\ of\ the\ number\ of\ electrons\ due\ to\ noise\ per\ pixel$]. It mainly arise from the on-chip preamplifier.

- dark current: noise arising from the stochastic thermal generation of electrons within the CCD structure, units [$number\ of\ electrons/pixel/s$].

- quantum efficiency: mean fraction of photons converted into electrons by a pixel per incoming photon. A value of 1 indicates all incoming photons are converted into electrons, a value of 0.5 indicates half of them are converted into electrons and a value of 0 indicates none of them are converted into electrons.

- gain: mean number of electrons generated per photon absorbed per pixel
- pixel size: width (or height) of a physical pixel from the camera, units [μm]. The pixel aspect ratio is always one.
- pixel number: number of pixels in the camera along a single dimension. The horizontal and vertical axis of the camera both have the same number of pixels.

Optical Parameters

- numerical aperture: quantity describing the ability of the microscope's objective to gather light from the specimen. A high numerical aperture (>1) allows to visualize fine specimen details. The resolution of an optical system increases – i.e. the minimal distance for distinguishing two nearby fluorophores decreases – as the numerical aperture increases according to the following equation:

$$d_{min} = \frac{wavelength}{2 \times numerical\ aperture}$$

- wavelength: the wavelength of light emitted by the fluorophore, units [nm]
- magnification: ratio between the specimen size as viewed by the camera and as in the sample.

The Launch panel is subdivided into three components: a *stack* and a *run button*, a *show panel* that includes a *tutorial* and a *simulator checkbox*, a *distributions popupmenu* and an *examples popupmenu*.
The *stack button* launches the simulation based on the simulation parameters listed above to generate an image sequence of blinking emitters whose two-dimensional spatial distribution is shown in the *start menu* (Fig. 2) under the name *Preview*. A *zoom button* displays the *Preview* on a larger window. Once the stack of images is generated, the *stack button* is disabled and the *run button* launches the *simulator* and *tutorial menu* depending on which *checkbox* is ticked in the *show panel*. The user may save the generated image stack and corresponding parameters with the *save button*. Saved image stacks can be re-loaded in the GUI using the *load button*. The *tutorial menu* only requires the "2D SOFI" algorithm to run before opening. Prior to launching the *simulator menu*, the *start menu* first opens an *algorithms* window from which the user may select one out of two different SOFI algorithms and one out of two different STORM algorithms which are listed below:

- 2D SOFI: SOFI algorithm as described in the *tutorial menu* (Fig. 3) for two dimensional image sequences
- 2D SOFI GPU: "2D SOFI" algorithm whose cross-cumulants are computed with Matlab CUDA-enabled GPU to decrease the algorithm's computation time
- STORM: Localization algorithm developed by Geissbuehler et al (2011)

- FalconSTORM GPU: STORM algorithm developed by Unser et al (2014) using Matlab CUDA-enabled GPU computing.

Please refer to README.txt for operating the CUDA-enabled NVIDIA GPU algorithms.

The *distribution popupmenu* allows the user to define the two-dimensional pattern according which the fluorophores will be distributed in space. The items – or modes – the user may select are listed below:

- random: the fluorophores are uniformly distributed throughout the sample plane. In this mode, the *text* and *scroll bar* displayed near the *preview* are disabled.
- two emitters: two fluorophores are aligned and separated by a distance d. In this mode, the physical distance between the two fluorophores is specified below the *preview* and adjustable using the vertical *scroll bar* that lies near.
- circular: the fluorophores are uniformly distributed within a randomly-placed disk. In this mode, the number of disks is specified below the *preview* and adjustable using the vertical *scroll bar.*
- annular: the fluorophores are uniformly distributed within a randomly-placed annulus. In this mode, the number of annulus is specified below the *preview* and adjustable using the vertical *scroll bar.*
- segment: the fluorophores are uniformly distributed within a randomly-placed rectangle. In this mode, the number of rectangles is specified below the *preview* and adjustable using the vertical *scroll bar.*
- siemens star: the fluorophores are uniformly distributed within a siemens star pattern. In this mode, the frequency (i.e number of branches) of the star pattern is specified below the *preview* and adjustable using the vertical *scroll bar.*
- user defined: the fluorophores are uniformly distributed within a binary image provided by the user. In this mode, the vertical *scroll bar* is disabled and the size of the field of view is fixed according to the input image. If the image provided by the user is not binary, the software will convert into a binary image by setting half of the pixels to 1, namely those with the highest GSV (grayscale value), and the other half to 0. In addition, if the provided image is not 2-dimensional, it will be replaced by a 2D *random distribution.* Furthermore, if the number of emitters provided by the user in the *start menu* exceeds the dimensions of the object, the number of emitters will be reduced accordingly to fit the dimensions of the object. Finally if the provided image do exhibit the dimensions of a square, part of the image will be truncated accordingly. It is expected from the user to choose a binary image describing the signal by white pixels and the background by black pixels.

The *return button* ↩ displayed next to the *launch panel* allows the user to display the *tutorial* and the *simulator menu* (depending on which *checkbox* from the *show panel* is ticked) with the most recent datasets. If nothing is checked, the *return button* is disabled.

2.2 Tutorial menu

The purpose of the *tutorial* is to visualize the SOFI processing steps from the image sequence acquisition to the final SOFI image (including a balanced SOFI analysis). This interface was mainly designed for tutorial purposes.

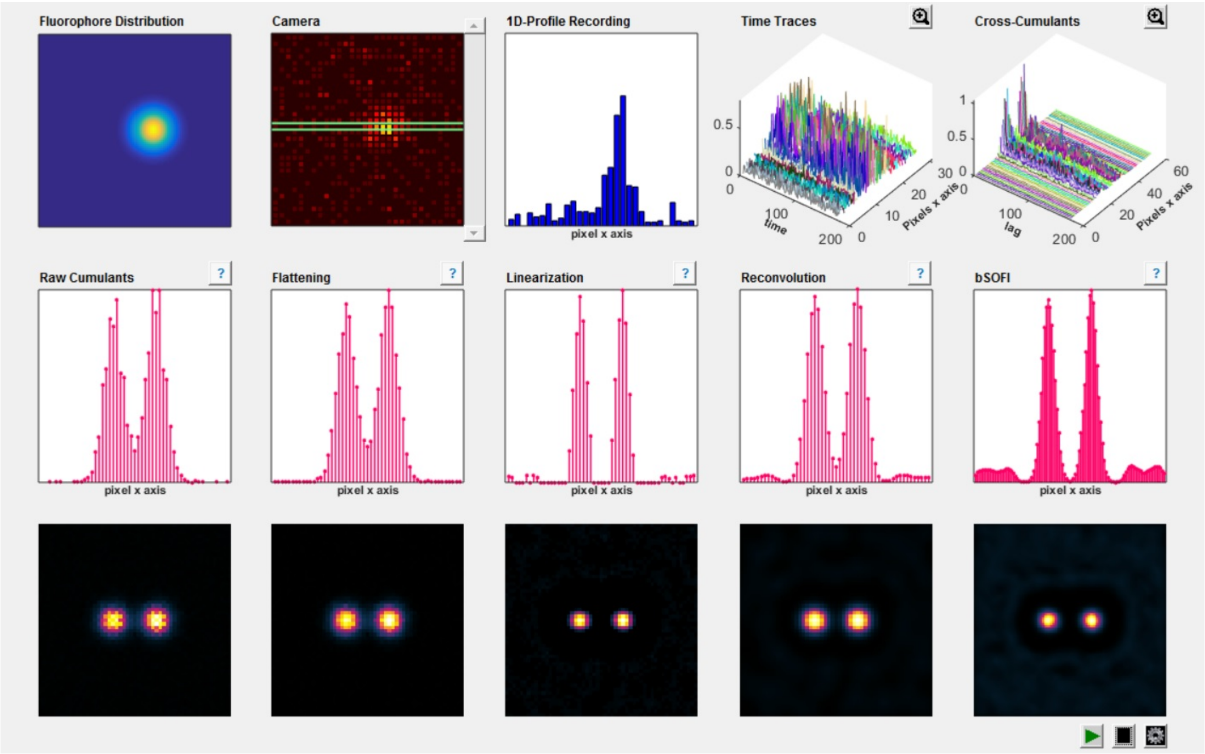

Figure 3: Tutorial menu explaining all the image processing steps of the SOFI algorithm.

The windows along with a short description are listed below:

- The *Fluorophore Distribution* window displays an image sequence of two blinking fluorophores. The colormap is defined in order to set high and low intensity values to white and dark-blue respectively.
- The *Camera* window displays each pixel from the detector grid – delimited by straigth dark lines – and its corresponding intensity value. The colormap is defined in order to set high intensity pixels to white and yellow whereas low intensity pixels to black and dark red. Two horizontal green lines encompasses a single row of pixels, thereafter named *pixel row*, and can be displaced from top to bottom with the vertical slider nearby.
- The *1D-Profile Recording* window displays the intensity values recorded by the *pixel row* of each frame sequentially.
- The *Time Traces* window displays the intensities as animated lines, each corresponding to the value of a single pixel within the *pixel row*, plotted against time (units in $[number\ of\ frames]$).

- The *Cross-Cumulants* window displays the 2nd order cross-cumulant pixels of the intensity time traces displayed in the previous window.

- The *Raw Cumulants* windows display the 2nd order Cross-Cumulants of the pixel time traces at zero time lag, $\tau = 0$ in both the one- (top window) and two- (bottom window) dimensional case.

- *Flattening* depicts the flattened 2nd order cross-cumulants from the *Raw Cumulants* window.

- *Linearization* depicts the linearized and flattened 2nd order cross-cumulants.

- *Reconvolution* depicts the convolved, linearized and flattened 2nd order cross-cumulants defined thereafter as the 2nd order SOFI image.

- *bSOFI* depicts the balanced SOFI image determined using the 3rd and 4th order SOFI images.

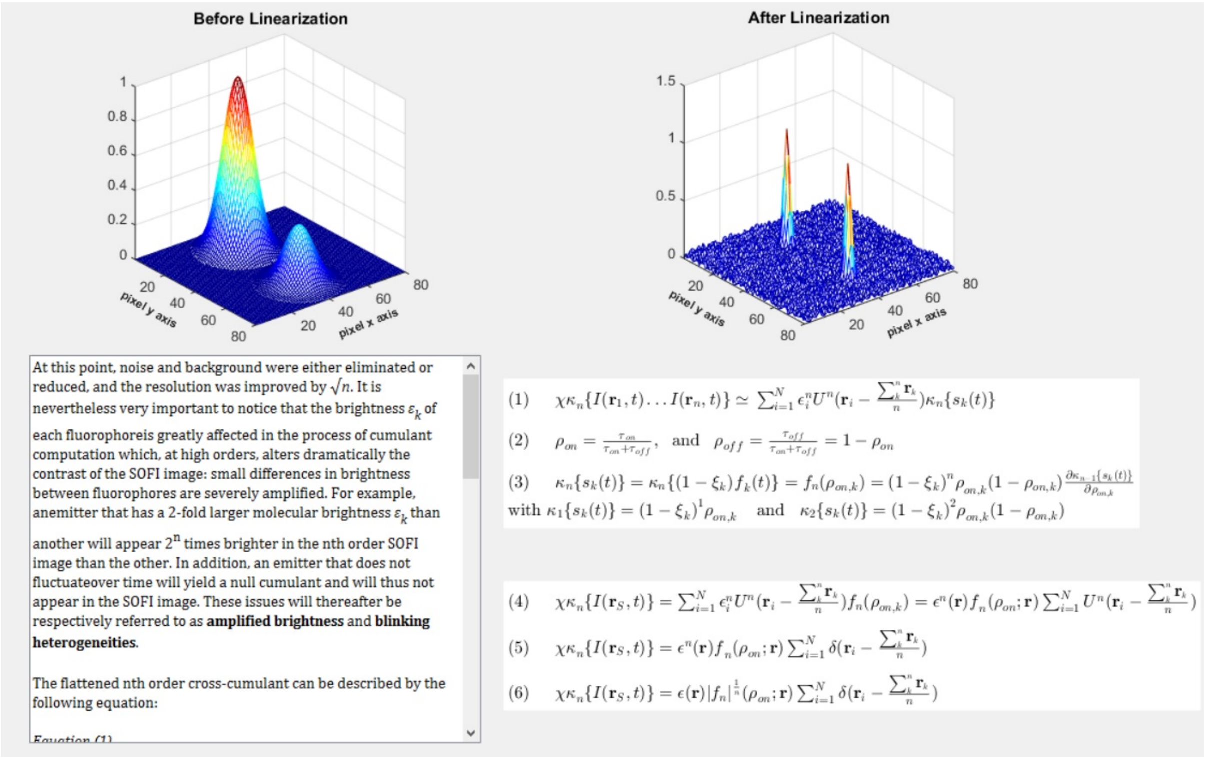

Figure 4: Help Menu explaining the linearization procedure of the SOFI algorithm.

The *tutorial menu* also allows the user to visualize detailed explanations with additional supporting figures and equations of each SOFI processing steps by interacting with the *help buttons* . Figure 4 displays the *help menu* from the *Linearization* step as an example.

Play, *stop* and *zoom buttons* are included in the *tutorial menu menu* in order to respectively start, stop and zoom over the animations presented in the five upper windows.

To conclude, a *settings button* readdresses the user towards the *start menu* (Fig. 2).

2.3 Simulator menu

The *simulator* allows a qualitative assessment i.e. how the different n-order SOFI images may compare to the true simulated distribution of fluorophores, their widefield image and the stochastic optical reconstruction (STORM) image. This interface is designed for using SOFI and to simulate qualitatively the influence of optical parameters and sample characteristics when performing SOFI imaging.

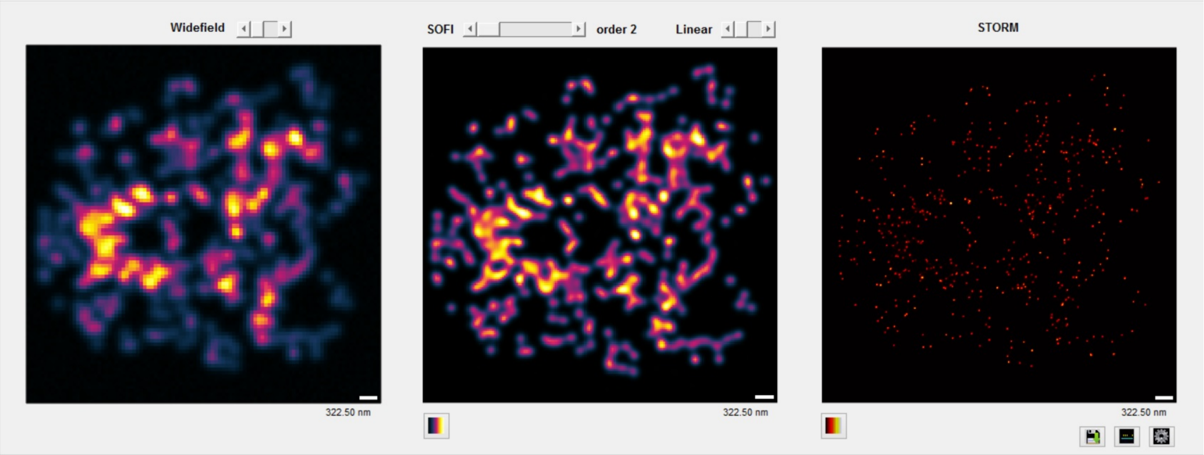

Figure 5: Simulator menu showing (from left to right) widefield, SOFI images of different orders, and STORM image.

Figure 5 displays the widefield microscopy image or the true distribution of fluorophores (not shown here; white dots over a dark background) available for viewing by using the slider above.

The middle figure presents a SOFI image which the user can select from a list comprising cumulant orders up to seven, including the balanced SOFI representation, by using the slider above. Another slider on the top right corner allows the user to visualize either the linearized cumulant – SOFI image – or the raw – unprocessed – cumulant. A *dynamic range button* for adjusting the contrast is provided both for the SOFI image and the STORM image.

A *scale button* enables or disables the scale bars displayed on the bottom right corner of each figure.

The user may save all images from the *simulator menu* in uncompressed TIFF formats using the *save button*.

To conclude, a *settings button* readdresses the user towards the *start menu* described in section 2.1.

3 Appendix

The experimental set-up parameters of the *reference example* provided in the *start menu* (described in section 2 and referred to "standard conditions" or "reference") are listed in the table below:

**Parameter**	**Value**	**Parameter**	**Value**
density in sample	3.6 $/\mu m^2$	numerical aperture	1.3
number on camera	150	magnification	100
acquisition time	100 s	acquisition speed	100 $/s$
signal per frame	400 $photons$	readout noise	1.6 rms
on-state lifetime	20 ms	dark current	0.06 $e^-/pixel/s$
off-state lifetime	180 ms	quantum efficiency	0.7
average bleaching time	80 ms	gain	6
background	4 $photons$	pixel size	6.45 μm
wavelength	600 nm	pixel number	100
