# Supplementary material for: SOFI Simulation Tool: A Software Package for Simulating and Testing Super-Resolution Optical Fluctuation Imaging
Source: PLoS One. 2016 Sep 1;11(9):e0161602. doi: 10.1371/journal.pone.0161602 (PMC5008722; doi:10.1371/journal.pone.0161602)
Supplement: S2 Appendix — Zip file which includes the software package. The software is written in MATLAB, equipped with graphical user interface and freely available together with a user manual also at [16]. (ZIP) [file pone.0161602.s002.zip › sofisimulationtool-2016-07-12/GUI/codeUtils/help_TextFigures/axes10/axes10.docx]

The flattened n^th^ order cross-cumulant can be described by the following equation:

$${X\kappa}_{n}\left\{ I\left( \boldsymbol{r}_{\boldsymbol{S}},t \right) \right\}=\sum_{i=1}^{N} {\epsilon_{k}^{n}U}^{n}\left( \boldsymbol{r}_{i}-\frac{\sum_{k}^{n} \boldsymbol{r}_{k}}{n} \right)f_{n}\left( \rho_{on,k} \right)={\epsilon^{n}\left( \boldsymbol{r} \right)f}_{n}\left( \rho_{on};\boldsymbol{r} \right)\sum_{i=1}^{N} U^{n}\left( \boldsymbol{r}_{i}-\frac{\sum_{k}^{n} \boldsymbol{r}_{k}}{n} \right)$$

as shown in the tutorial step *Linearization*.

Since neither $\epsilon(\boldsymbol{r)}$ nor $\rho_{on}$ vary with the order of the cumulant *n*, combining several cumulants allowed us to determine the molecular density $N\left( \vec{r} \right)$, the spatial distribution of molecular brightness $\epsilon\left( \vec{r} \right)\approx\sum\epsilon_{k}$ and the on-time ratio map $\rho_{on}\left( \vec{r} \right)\approx\sum\rho_{on,k}$ of fluorophores each with its own level of accuracy ($\rho_{on}$ having the highest) as described in S. Geissbuehler et al. 2012.

The n^th^ order linearized cumulants are proportional to $f_{n}$ which has been shown in the tutorial step *Linearization* to be a polynomial of order *n*, thus having *n* roots for $\rho_{on}$. In other words, after brightness linearization, $\left| f_{n} \right|^{\frac{1}{n}}$ introduces artifacts in the resulting image due to its zeros. However, using the on-ratio map $\rho_{on}$, we can easily determine where $\left| f_{n} \right|^{\frac{1}{n}}$ will drop towards zero and fill these locations with the (n-1)th order linearized cumulants (instead of the actual nth order one). Indeed, according to Figure 1, $\left| f_{n} \right|^{\frac{1}{n}}$ zeros always correspond to high $\left| f_{n-1} \right|^{\frac{1}{n-1}}$ values.

Correction for this artefact is introduced in equation (2) to yield the bSOFI image that can be roughly approximated by:

$${X\kappa}_{n}\left\{ I\left( \boldsymbol{r}_{1},t \right)\ldots I\left( \boldsymbol{r}_{n},t \right) \right\}\approx\epsilon\left( \boldsymbol{r} \right)\sum_{i=1}^{N} U\left( \frac{\boldsymbol{r}_{i}-\frac{\sum_{k}^{n} \boldsymbol{r}_{k}}{n}}{n} \right)$$

Equation (2):

$${X\kappa}_{n}\left\{ I\left( \boldsymbol{r}_{1},t \right)\ldots I\left( \boldsymbol{r}_{n},t \right) \right\}={\epsilon\left( \boldsymbol{r} \right)\left| f_{n} \right|}^{\frac{1}{n}}\left( \rho_{on};\boldsymbol{r} \right)\sum_{i=1}^{N} U\left( \frac{\boldsymbol{r}_{i}-\frac{\sum_{k}^{n} \boldsymbol{r}_{k}}{n}}{n} \right)$$
