# Supplementary material for: SOFI Simulation Tool: A Software Package for Simulating and Testing Super-Resolution Optical Fluctuation Imaging
Source: PLoS One. 2016 Sep 1;11(9):e0161602. doi: 10.1371/journal.pone.0161602 (PMC5008722; doi:10.1371/journal.pone.0161602)
Supplement: S2 Appendix — Zip file which includes the software package. The software is written in MATLAB, equipped with graphical user interface and freely available together with a user manual also at [16]. (ZIP) [file pone.0161602.s002.zip › sofisimulationtool-2016-07-12/GUI/codeUtils/help_TextFigures/axes5 - not used/axes5.docx]

**2^nd^ order cross-cumulants of pixel time traces**

The intensity distribution measured over time on a detector pixel $\vec{r}$ of a sample composed of M independently fluctuating emitters can be written as:

$$I\left( \vec{r},t \right)=\sum_{k=1}^{M} \epsilon_{k}U\left( \vec{r}-\vec{r}_{k} \right)s_{k}\left( t \right)+b\left( \vec{r} \right)$$

Where$\epsilon_{k}$, $\vec{r}_{k}$ and $s_{k}\left( t \right)$ denote the emitter’s brightness, its position and its normalized temporal fluctuations. $U\left( \vec{r} \right)$ is the system’s point-spread function (PSF) and *b* represents a spatially-varying background term. Ideally, an infinite resolution and zero noise image would be fully characterized by the fluorophores’ brightness and positions:

$$I_{ideal}\left( \vec{r} \right)=\sum_{k=1}^{M} \epsilon_{k}\delta\left( \vec{r}-\vec{r}_{k} \right)$$

Therefore, we would like to design a system $\mathbb{S}$ such that:

$$\left. \lim_{N\to\infty} \mathbb{S}\left\{ I\left( \vec{r},t \right) \right\} \right|_{t\mathbb{\in R}}=I_{ideal}\left( \vec{r} \right)$$

With N a parameter of the system. $I\left( \vec{r},t \right)$ is in principle equal to the sum of $s_{k}\left( t \right)$, the time traces of each fluorophore – corrupted by shot and background noise– blurred by the PSF and recorded by the pixels of the detector – corrupted by thermal and readout noise. Therefore, our system$\mathbb{S}$ should remove shot, thermal and readout noise from $s_{k}\left( t \right)$, remove the spatially variant background $b\left( \vec{r} \right)$ and in the meantime reduce the PSF’s dimension to approach a $\delta$. In that case, we would in theory be able to generate superresolution images.

Using time – or more specifically, the fluctuating behavior of fluorophores – as a way to achieve these objectives relies on ***higher-order statistics***. Statiscal measures are generally known as either ***moments*** or ***cumulants***. The 1^st^ and 2^nd^ order moments are widely used in all engineering fields and are most commonly known as the mean and auto-correlation (variance) of stochastic processes.

The n^th^ order moment (n >= 4) of two statistically independent random processes (such as the amount of light emitted from two distinct fluorophores) does not equal the sum of the moments of the individual random processes:

$\mu_{n}\left( I\left( \vec{r_{1}},t \right)+I\left( \vec{r_{2}},t \right) \right)\neq\mu_{n}\left( I\left( \vec{r_{1}},t \right) \right)+\mu_{n}\left( I\left( \vec{r_{2}},t \right) \right)$ for $n\geq4$

In other words, the n^th^ order moment of $I\left( \vec{r},t \right)$ would result mainly from cross-talks between the two fluorophores. This principle can be further extended resulting in many more cross-talks as the number of fluorophore in $I\left( \vec{r},t \right)$ is increasing. This prevents using higher-order moments for designing $\mathbb{S}$.

One way to resolve this issue is to compute ***cumulants*** rather than *moments.* A rigorous analytical description of cumulants is beyond the scope of this tutorial and we will thus only list a few of its key properties and explain how they enable to provide noise and background free superresolution.

Defintion: The nth-order cumulant of random variables $\boldsymbol{x}=\left( x_{1},\ldots,x_{n} \right)$ is defined as the coefficient of $\boldsymbol{v}=\left( v_{1},\ldots,v_{n} \right)$ in the Taylor series expansion of the cumulant generating function:

$$K\left( \boldsymbol{v} \right)=lnE\left\{ e^{j\left\langle\boldsymbol{v},\boldsymbol{x} \right\rangle} \right\}$$

To compare, the moment generating function is the following:

$$M\left( \boldsymbol{v} \right)=E\left\{ e^{j\left\langle\boldsymbol{v},\boldsymbol{x} \right\rangle} \right\}$$

Therefore, the nth-order cumulants can be inferred in terms of its joint moments of orders up to n as follows:

$$C_{x}\left( I \right)=\sum_{U_{p=1}^{q}I_{p}=I} \left( -1 \right)^{q-1}\left( q-1 \right)!\prod_{p=1}^{q} m_{x}\left( I_{p} \right)$$

Where $U_{p=1}^{q}I_{p}=I$ denotes summation over all partitions of set *I*. See *Table A1 from Mendel et al (1991).*

Property 1: If $\lambda_{i}$, $i=1,\ldots,n,$ are constants, and $x_{i}$, $i=1,\ldots,n,$ are random variables, then

$$\kappa_{n}\left( \lambda_{1}x_{1},\ldots,\lambda_{k}x_{n} \right)=\left( \prod_{i=1}^{n} \lambda_{i} \right)\kappa_{n}\left( x_{1},\ldots,x_{n} \right)$$

Property 2: If $\alpha$ is a constant, then

$$\kappa_{n}\left( {\alpha+x}_{1},\ldots,x_{n} \right)=\kappa_{n}\left( x_{1},\ldots,x_{n} \right)$$

Property 3: If the random variable $\left\{ x_{i} \right\}$ are indepedent of the random variables $\left\{ y_{i} \right\}, i=1,\ldots,n$ then

$$\kappa_{n}\left( x_{1}+y_{1},\ldots,x_{n}+y_{n} \right)\boldsymbol{=}\kappa_{n}\left( x_{1},\ldots,x_{n} \right)+\kappa_{n}\left( y_{1},\ldots,y_{n} \right)$$

As explained previsouly, cross-terms (cross-talks between many fluorophores) are present in moments and absent in cumulants. This difference is formulated by property 3 which is exclusive to cumulants (can be easily demonstrated using « Definition »).

Returning to the equation of $I\left( \vec{r},t \right)$, the intensity distribution measured over time on a detector pixel $\vec{r}$ of a sample composed of M independetly fluctuating emitters can be written as:

$$I\left( \vec{r},t \right)=\sum_{k=1}^{M} \epsilon_{k}U\left( \vec{r}-\vec{r}_{k} \right)s_{k}\left( t \right)+b\left( \vec{r} \right)$$

The n^th^ order cumulants is described by:

$$\kappa_{n}\left\{ I\left( \vec{r},t \right) \right\}\left( \boldsymbol{\tau} \right)=\kappa_{n}\left\{ \sum_{k=1}^{M} \epsilon_{k}U\left( \vec{r}-\vec{r}_{k} \right)s_{k}\left( t \right)+b\left( \vec{r} \right) \right\}\left( \boldsymbol{\tau} \right)\underset{\Rightarrow}{Prop. 2}\kappa_{n}\left\{ \sum_{k=1}^{M} \epsilon_{k}U\left( \vec{r}-\vec{r}_{k} \right)s_{k}\left( t \right) \right\}\left( \boldsymbol{\tau} \right)$$

$$\underset{\Rightarrow}{Prop. 3}\sum_{k=1}^{M} \kappa_{n}\left\{ \epsilon_{k}U\left( \vec{r}-\vec{r}_{k} \right)s_{k}\left( t \right) \right\}\left( \boldsymbol{\tau} \right)\underset{\Rightarrow}{Prop. 1}\sum_{k=1}^{M} \epsilon_{k}^{n}U^{n}\left( \vec{r}-\vec{r}_{k} \right)\kappa_{n}\left\{ s_{k}\left( t \right) \right\}\left( \boldsymbol{\tau} \right)$$

$\epsilon_{k}^{n}U^{n}\left( \vec{r}-\vec{r}_{k} \right)$ contains information about the brightness $\epsilon_{k}^{2}$, location $\vec{r}_{k}$and induced point-spread function $U^{n}\left( \vec{r} \right)$ of the fluorophore $k$. It is straightforward to notice that the position of the fluorophore, as described in $I\left( \vec{r},t \right)$, is unchanged and thus $\kappa_{n}\left\{ I\left( \vec{r},t \right) \right\}$ does not affect the true location of fluorophores. We can also notice that the point-spread function $U\left( \vec{r}-\vec{r}_{k} \right)$ has been replaced by $U^{n}\left( \vec{r}-\vec{r}_{k} \right)$. If we approximate the point-spread function $U\left( \vec{r} \right)$ by a Gaussian function, we obtain:

$$U\left( \vec{r} \right)=e^{-\frac{r^{2}}{2\sigma^{2}}} and U^{n}\left( \vec{r} \right)=e^{-\frac{r^{2}}{2\left( \frac{\sigma}{\sqrt{n}} \right)^{2}}}$$

In that case, the point-spread function of $\kappa_{n}\left\{ I\left( \vec{r},t \right) \right\}$ becomes thinner by a factor $\sqrt{n}$ and as consequence, there is a resolution improvement of a factor $\sqrt{n}$ in $\kappa_{n}\left\{ I\left( \vec{r},t \right) \right\}$ as compared to $I\left( \vec{r},t \right)$. In addition, due to Property 2, the spatially-varying background was effectively removed. Finally, since $\kappa_{n}\left\{ s_{k}\left( t \right) \right\}\left( \boldsymbol{\tau} \right)$ is neither necessary for resolution improvement or background suppression, we arbitrarily compute $\kappa_{n}\left\{ I\left( \vec{r},t \right) \right\}\left( \boldsymbol{0} \right)$ for an impressively fast computation (3-4 seconds for $n=\left\{ 1,2,3,4,5,6,7 \right\}$).

However, shot noise which is intrinsically in $s_{k}\left( t \right)$ (thus in $\kappa_{n}\left\{ s_{k}\left( t \right) \right\}$) and any other time-varying noise are not successfully suppressed by the approach we have introduced. In addition, as we increase the order of the cumulant, even though the PSF gets thinner, the number of pixels stays the same. This results in a loss of details in the shape of the “new” thinner PSF. Finally, as the resolution keeps increasing ($n$ increasing), a single pixel could eventually have several extremely thin PSF which we won’t be able to distinguish simply due to the fact that we cannot attribute a new pixel to each newly found PSFs. // fulfil the Nyquist sampling theorem

Therefore, there are two main challenges that should be addressed at this point:

- Suppress shot noise
- Compute inter-pixels values as the order of cumulant increases

The cumulants $\kappa_{n}\left\{ I\left( \vec{r},t \right) \right\}(\boldsymbol{0})$ depicted above are computed for each pixel detector individually. Nevertheless, it is also possible to combine the information of many pixels to effectively remove time-varying noise and determine inter-pixels values by computing ***cross-cumulants*** ${X\kappa}_{n}\left\{ I\left( \vec{r}_{1},t \right),\ldots,I\left( \vec{r}_{n},t \right) \right\}(\boldsymbol{0})$. In this case, the inter-pixel positions $\vec{r}$ are defined by $\vec{r}=\frac{\vec{r}_{1}+\ldots+\vec{r}_{n}}{n}$.

For the purpose of this tutorial, we will present how a background-and-noise-free superresolution image is generated with *2^nd^ order cross-cumulants*:

$${X\kappa}_{2}\left\{ I\left( \vec{r}_{1},t \right),I\left( \vec{r}_{2},t \right) \right\}\left( \tau\right)=E\left\{ I\left( \vec{r_{1}},t \right)I\left( \vec{r_{2}},t+\tau\right) \right\}=U\left( \frac{\vec{r}_{1}-\vec{r}_{2}}{\sqrt{2}} \right)\sum_{k=1}^{M} \epsilon_{k}^{2}U^{2}\left( \frac{\vec{r}_{1}+\vec{r}_{2}}{2}-\vec{r}_{k} \right)\left\langle s_{k}\left( t \right),s_{k}\left( t+\tau\right) \right\rangle_{t}$$

To compare: $\kappa_{2}\left\{ I\left( \vec{r_{1}},t \right) \right\}\left( \tau\right)=E\left\{ I\left( \vec{r_{1}},t \right)I\left( \vec{r_{1}},t+\tau\right) \right\}=\sum_{k=1}^{M} \epsilon_{k}^{2}U^{2}\left( \vec{r_{1}}-\vec{r}_{k} \right)\left\langle s_{k}\left( t \right),s_{k}\left( t+\tau\right) \right\rangle_{t}$

assuming a Gaussian point-spread function. As shown in the equation above, ${X\kappa}_{2}$ can be easily computed since it equals the temporal cross-correlation of $I\left( \vec{r_{1}},t \right)$ with $I\left( \vec{r_{2}},t \right)$. Since time-varying noise, including shot noise, is spatially and temporally uncorrelated (noise varies randomly with time and position), the temporal cross-correlation function of noise tends to zero. In addition, the thinner point spread function $U^{2}$ is now centered at $\vec{r}=\frac{\vec{r}_{1}+\vec{r}_{2}}{2}$ instead of $\vec{r_{1}}$, and thus a virtual pixel $\vec{r}$ between $\vec{r}_{1}$ and $\vec{r}_{2}$is effectively computed.

We could even further push the reasoning as such: the pixel at location $\vec{r}=\frac{\vec{r}_{1}+\vec{r}_{2}}{2}$ can also be computed with two other neighbouring pixels (i.e. in vertical direction rather than horizontal) $\vec{r}=\frac{\vec{r}_{3}+\vec{r}_{4}}{2}$. We could therefore calculate the pixel value in two different ways (with 2^nd^ order cross-cumulants) and then average them reducing drastically the amount of noise in the new pixel value. We can extend the reasoning to n^th^ order cross-cumulants where we would need *n* pixels for the computation and thus have a much higher number of ways of computing the cumulant pixel and averaging them all to even further reduce noise.

The resulting n^th^ order cross-cumulant can thus be described by the following equation:

$${X\kappa}_{n}\left\{ I\left( \vec{r},t \right) \right\}=\prod_{j<l}^{n} U\left( \frac{r_{j}{-r}_{l}}{\sqrt{n}} \right)\sum_{i=1}^{N} {\epsilon_{k}^{n}U}^{n}\left( r_{i}-\frac{\sum_{k}^{n} r_{k}}{n} \right)\kappa_{n}\left\{ s_{k}\left( t \right) \right\}$$

Figure 1 shows the comparison of ${X\kappa}_{2}\left\{ I\left( \vec{r}_{1},t \right),I\left( \vec{r}_{2},t \right) \right\}\left( \boldsymbol{0} \right)$ with a spline interpolation of $I\left( \vec{r},t_{0} \right)$. Fluorophores are seen “sharper” (due to a thinner point spread function) on ${X\kappa}_{2}\left\{ I\left( \vec{r}_{1},t \right),I\left( \vec{r}_{2},t \right) \right\}\left( \boldsymbol{0} \right)=E\left\{ I\left( \vec{r},t \right)I\left( \vec{r},t+\tau\right) \right\}(\tau=0)$ than on $I\left( \vec{r},{t=t}_{0} \right)$. In addition, in ${X\kappa}_{2}$, noise has been completely removed from $I\left( \vec{r},t \right)$. Figure 2 and 3 helps understanding why only considering ${X\kappa}_{2}\left\{ I\left( \vec{r}_{1},t \right),I\left( \vec{r}_{2},t \right) \right\}\left( \boldsymbol{0} \right)$ and not ${X\kappa}_{2}\left\{ I\left( \vec{r}_{1},t \right),I\left( \vec{r}_{2},t \right) \right\}\left( \tau\right), \tau\in\mathbb{R}^{+}$ or $\int{X\kappa}_{2}\left\{ I\left( \vec{r}_{1},t \right),I\left( \vec{r}_{2},t \right) \right\}\left( \tau\right)d\tau$ is appropriate. Indeed, since there are no practical gain in reducing noise and improving the resolution, computing only ${X\kappa}_{2}(0)$ is sufficient. Only the first few coefficients $\left( \tau\sim0 \right)$ provide sufficient information: ${X\kappa}_{2}\left( \tau\right)$ rapidly decays toward zero as $\tau$ increases.
