# Supplementary material for: SOFI Simulation Tool: A Software Package for Simulating and Testing Super-Resolution Optical Fluctuation Imaging
Source: PLoS One. 2016 Sep 1;11(9):e0161602. doi: 10.1371/journal.pone.0161602 (PMC5008722; doi:10.1371/journal.pone.0161602)
Supplement: S2 Appendix — Zip file which includes the software package. The software is written in MATLAB, equipped with graphical user interface and freely available together with a user manual also at [16]. (ZIP) [file pone.0161602.s002.zip › sofisimulationtool-2016-07-12/GUI/codeUtils/help_TextFigures/axes5 - not used/axes5_equations.docx]

(1) $I\left( \vec{r},t \right)=\sum_{k=1}^{M} \epsilon_{k}U\left( \vec{r}-\vec{r}_{k} \right)s_{k}\left( t \right)+b\left( \vec{r} \right)$

(2) $I_{ideal}\left( \vec{r} \right)=\sum_{k=1}^{M} \epsilon_{k}\delta\left( \vec{r}-\vec{r}_{k} \right)$

(3) $\left. \lim_{N\to\infty} \mathbb{S}\left\{ I\left( \vec{r},t \right) \right\} \right|_{t\mathbb{\in R}}=I_{ideal}\left( \vec{r} \right)$

(4) $\mu_{n}\left( I\left( \vec{r_{1}},t \right)+I\left( \vec{r_{2}},t \right) \right)\neq\mu_{n}\left( I\left( \vec{r_{1}},t \right) \right)+\mu_{n}\left( I\left( \vec{r_{2}},t \right) \right)$ for $n\geq4$

(5) $C_{x}\left( I \right)=\sum_{U_{p=1}^{q}I_{p}=I} \left( -1 \right)^{q-1}\left( q-1 \right)!\prod_{p=1}^{q} m_{x}\left( I_{p} \right)$

Cumulant Prop. 1: If $\lambda_{i}$, $i=1,\ldots,n,$ are constants, and $x_{i}$, $i=1,\ldots,n,$ are random variables, then

$$\kappa_{n}\left( \lambda_{1}x_{1},\ldots,\lambda_{k}x_{n} \right)=\left( \prod_{i=1}^{n} \lambda_{i} \right)\kappa_{n}\left( x_{1},\ldots,x_{n} \right)$$

Cumulant Prop. 2: If $\alpha$ is a constant, then

$$\kappa_{n}\left( {\alpha+x}_{1},\ldots,x_{n} \right)=\kappa_{n}\left( x_{1},\ldots,x_{n} \right)$$

Cumulant Prop. 3: If the random variable $\left\{ x_{i} \right\}$ are indepedent of the random variables $\left\{ y_{i} \right\}, i=1,\ldots,n$ then

$$\kappa_{n}\left( x_{1}+y_{1},\ldots,x_{n}+y_{n} \right)\boldsymbol{=}\kappa_{n}\left( x_{1},\ldots,x_{n} \right)+\kappa_{n}\left( y_{1},\ldots,y_{n} \right)$$

The n^th^ order cumulants is described by:

(6) $\kappa_{n}\left\{ I\left( \vec{r},t \right) \right\}\left( \boldsymbol{\tau} \right)=\kappa_{n}\left\{ \sum_{k=1}^{M} \epsilon_{k}U\left( \vec{r}-\vec{r}_{k} \right)s_{k}\left( t \right)+b\left( \vec{r} \right) \right\}\left( \boldsymbol{\tau} \right)\underset{\Rightarrow}{Prop. 2}\kappa_{n}\left\{ \sum_{k=1}^{M} \epsilon_{k}U\left( \vec{r}-\vec{r}_{k} \right)s_{k}\left( t \right) \right\}\left( \boldsymbol{\tau} \right)$

$$\underset{\Rightarrow}{Prop. 3}\sum_{k=1}^{M} \kappa_{n}\left\{ \epsilon_{k}U\left( \vec{r}-\vec{r}_{k} \right)s_{k}\left( t \right) \right\}\left( \boldsymbol{\tau} \right)\underset{\Rightarrow}{Prop. 1}\sum_{k=1}^{M} \epsilon_{k}^{n}U^{n}\left( \vec{r}-\vec{r}_{k} \right)\kappa_{n}\left\{ s_{k}\left( t \right) \right\}\left( \boldsymbol{\tau} \right)$$

(7) ${X\kappa}_{2}\left\{ I\left( \vec{r}_{1},t \right),I\left( \vec{r}_{2},t \right) \right\}\left( \tau\right)=E\left\{ I\left( \vec{r_{1}},t \right)I\left( \vec{r_{2}},t+\tau\right) \right\}=U\left( \frac{\vec{r}_{1}-\vec{r}_{2}}{\sqrt{2}} \right)\sum_{k=1}^{M} \epsilon_{k}^{2}U^{2}\left( \frac{\vec{r}_{1}+\vec{r}_{2}}{2}-\vec{r}_{k} \right)\left\langle s_{k}\left( t \right),s_{k}\left( t+\tau\right) \right\rangle_{t}$

(8) $\kappa_{2}\left\{ I\left( \vec{r_{1}},t \right) \right\}\left( \tau\right)=E\left\{ I\left( \vec{r_{1}},t \right)I\left( \vec{r_{1}},t+\tau\right) \right\}=\sum_{k=1}^{M} \epsilon_{k}^{2}U^{2}\left( \vec{r_{1}}-\vec{r}_{k} \right)\left\langle s_{k}\left( t \right),s_{k}\left( t+\tau\right) \right\rangle_{t}$

(9) $\kappa_{n}\left\{ I\left( \vec{r},t \right) \right\}=\prod_{j<l}^{n} U\left( \frac{r_{j}{-r}_{l}}{\sqrt{n}} \right)\sum_{i=1}^{N} {\epsilon_{k}^{n}U}^{n}\left( r_{i}-\frac{\sum_{k}^{n} r_{k}}{n} \right)\kappa_{n}\left\{ s_{k}\left( t \right) \right\}$

(1) $I\left( \boldsymbol{r},t \right)=\sum_{k=1}^{M} \epsilon_{k}U\left( \boldsymbol{r}-\boldsymbol{r}_{k} \right)s_{k}\left( t \right)+b\left( \boldsymbol{r} \right)$

(2) $I_{ideal}\left( \boldsymbol{r} \right)=\sum_{k=1}^{M} \epsilon_{k}\delta\left( \boldsymbol{r}-\boldsymbol{r}_{k} \right)$

(3) $\left. \lim_{N\to\infty} \mathbb{S}\left\{ I\left( \boldsymbol{r},t \right) \right\} \right|_{t\mathbb{\in R}}=I_{ideal}\left( \boldsymbol{r} \right)$

(4) $\mu_{n}\left( I\left( \boldsymbol{r}_{\boldsymbol{1}},t \right)+I\left( \boldsymbol{r}_{2},t \right) \right)\neq\mu_{n}\left( I\left( \boldsymbol{r}_{\boldsymbol{1}},t \right) \right)+\mu_{n}\left( I\left( \boldsymbol{r}_{\boldsymbol{1}},t \right) \right)$ for $n\geq4$

(5) $C_{x}\left( I \right)=\sum_{U_{p=1}^{q}I_{p}=I} \left( -1 \right)^{q-1}\left( q-1 \right)!\prod_{p=1}^{q} m_{x}\left( I_{p} \right)$

Cumulant Prop. 1: If $\lambda_{i}$, $i=1,\ldots,n,$ are constants, and $x_{i}$, $i=1,\ldots,n,$ are random variables, then

$$\kappa_{n}\left( \lambda_{1}x_{1},\ldots,\lambda_{k}x_{n} \right)=\left( \prod_{i=1}^{n} \lambda_{i} \right)\kappa_{n}\left( x_{1},\ldots,x_{n} \right)$$

Cumulant Prop. 2: If $\alpha$ is a constant, then

$$\kappa_{n}\left( {\alpha+x}_{1},\ldots,x_{n} \right)=\kappa_{n}\left( x_{1},\ldots,x_{n} \right)$$

Cumulant Prop. 3: If the random variable $\left\{ x_{i} \right\}$ are indepedent of the random variables $\left\{ y_{i} \right\}, i=1,\ldots,n$ then

$$\kappa_{n}\left( x_{1}+y_{1},\ldots,x_{n}+y_{n} \right)\boldsymbol{=}\kappa_{n}\left( x_{1},\ldots,x_{n} \right)+\kappa_{n}\left( y_{1},\ldots,y_{n} \right)$$

(6) $\kappa_{n}\left\{ I\left( \vec{r},t \right) \right\}\left( \boldsymbol{\tau} \right)=\kappa_{n}\left\{ \sum_{k=1}^{M} \epsilon_{k}U\left( \vec{r}-\vec{r}_{k} \right)s_{k}\left( t \right)+b\left( \vec{r} \right) \right\}\left( \boldsymbol{\tau} \right)\underset{\Rightarrow}{Prop. 2}\kappa_{n}\left\{ \sum_{k=1}^{M} \epsilon_{k}U\left( \vec{r}-\vec{r}_{k} \right)s_{k}\left( t \right) \right\}\left( \boldsymbol{\tau} \right)$

$$\underset{\Rightarrow}{Prop. 3}\sum_{k=1}^{M} \kappa_{n}\left\{ \epsilon_{k}U\left( \vec{r}-\vec{r}_{k} \right)s_{k}\left( t \right) \right\}\left( \boldsymbol{\tau} \right)\underset{\Rightarrow}{Prop. 1}\sum_{k=1}^{M} \epsilon_{k}^{n}U^{n}\left( \vec{r}-\vec{r}_{k} \right)\kappa_{n}\left\{ s_{k}\left( t \right) \right\}\left( \boldsymbol{\tau} \right)$$

(7) ${X\kappa}_{2}\left\{ I\left( \boldsymbol{r}_{1},t \right),I\left( \boldsymbol{r}_{2},t \right) \right\}\left( \tau\right)=E\left\{ I\left( \boldsymbol{r}_{1},t \right)I\left( \boldsymbol{r}_{2},t+\tau\right) \right\}=U\left( \frac{\boldsymbol{r}_{1}-\boldsymbol{r}_{2}}{\sqrt{2}} \right)\sum_{k=1}^{M} \epsilon_{k}^{2}U^{2}\left( \frac{\boldsymbol{r}_{1}+\boldsymbol{r}_{2}}{2}-\boldsymbol{r}_{k} \right)\left\langle s_{k}\left( t \right),s_{k}\left( t+\tau\right) \right\rangle_{t}$

(8) $\kappa_{2}\left\{ I\left( \boldsymbol{r}_{1},t \right) \right\}\left( \tau\right)=E\left\{ I\left( \boldsymbol{r}_{1},t \right)I\left( \boldsymbol{r}_{1},t+\tau\right) \right\}=\sum_{k=1}^{M} \epsilon_{k}^{2}U^{2}\left( \boldsymbol{r}_{1}-\vec{r}_{k} \right)\left\langle s_{k}\left( t \right),s_{k}\left( t+\tau\right) \right\rangle_{t}$

(9) $\kappa_{n}\left\{ I\left( \boldsymbol{r},t \right) \right\}=\prod_{j<l}^{n} U\left( \frac{\boldsymbol{r}_{j}{-\boldsymbol{r}}_{l}}{\sqrt{n}} \right)\sum_{i=1}^{N} {\epsilon_{k}^{n}U}^{n}\left( \boldsymbol{r}_{i}-\frac{\sum_{k}^{n} \boldsymbol{r}_{k}}{n} \right)\kappa_{n}\left\{ s_{k}\left( t \right) \right\}$
