# Supplementary material for: SOFI Simulation Tool: A Software Package for Simulating and Testing Super-Resolution Optical Fluctuation Imaging
Source: PLoS One. 2016 Sep 1;11(9):e0161602. doi: 10.1371/journal.pone.0161602 (PMC5008722; doi:10.1371/journal.pone.0161602)
Supplement: S2 Appendix — Zip file which includes the software package. The software is written in MATLAB, equipped with graphical user interface and freely available together with a user manual also at [16]. (ZIP) [file pone.0161602.s002.zip › sofisimulationtool-2016-07-12/GUI/codeUtils/help_TextFigures/axes6/axes6.docx]

**Raw cumulants**

The sample is composed of M stochastically independently fluctuating emitters. The intensity distribution over time per pixel $\vec{r}$ can be written as

$$I\left( \vec{r},t \right)=\sum_{k=1}^{M} \epsilon_{k}U\left( \vec{r}-\vec{r}_{k} \right)s_{k}\left( t \right)+b\left( \vec{r} \right)$$

where $\epsilon_{k}$, $\vec{r}_{k}$ and $s_{k}\left( t \right)$ denote the brightness, position and normalized temporal fluctuations of the k^th^-emitter. $U\left( \vec{r} \right)$ is the point-spread function (PSF) and *b* represents a temporally constant background. Supposing an infinite resolution and zero noise contribution, we can write

$$I_{ideal}\left( \vec{r} \right)=\sum_{k=1}^{M} \epsilon_{k}\delta\left( \vec{r}-\vec{r}_{k} \right)$$

which represents the object. Ultimately, we would like to design an imaging system $\mathbb{S}$ such that in an ideal case, we would image perfectly the object

$$\lim_{N\to\infty} \mathbb{S}_{N}\left\{ I\left( \vec{r},t \right) \right\}=I_{ideal}\left( \vec{r} \right)$$

To approach this perfect imaging, we will take N images for achieving a “super-resolved image”. In fact, we exploit the temporal fluctuations registered as a sequence of individual images. $I\left( \vec{r},t \right)$ can be split in a spatial component $\epsilon_{k}U\left( \vec{r}-\vec{r}_{k} \right)$ and a temporal fluctuating component $s_{k}\left( t \right)$, the time traces of the k^th^-emitter. Ideal imaging fully exploits $s_{k}\left( t \right)$ to generate a super-resolved image.

Using time – or more specifically, the temporal fluctuations of emitters – as a way to achieve super-resolution can be performed by employing ***higher-order statistics***: the photon traces per pixel are analysed based on moments or cumulants . The 1^st^ and 2^nd^ order moments are widely used in engineering and are known as the mean and variance of a stochastic process.

The 4th and all higher order moments of two statistically independent stochastic processes does not equal the sum of the moments of the individual processes meaning moments are not additive:

$\mu_{n}\left( I\left( \vec{r_{1}},t \right)+I\left( \vec{r_{2}},t \right) \right)\neq\mu_{n}\left( I\left( \vec{r_{1}},t \right) \right)+\mu_{n}\left( I\left( \vec{r_{2}},t \right) \right)$ for $n\geq4$

In other words, the n^th^ order moment of $I\left( \vec{r},t \right)$ would result mainly from crosstalks between the two fluorophores. This principle can be further extended resulting in many more cross-talks as the number of fluorophore in $I\left( \vec{r},t \right)$ is increasing.

An elegant way to resolve this issue is to use ***cumulants*** rather than *moments.* A rigorous analytical description of cumulants is beyond the scope of this tutorial and we will thus only list a few of its key properties and explain how they enable to provide background-free super-resolution.

Defintion: The nth-order cumulant of random variables $\boldsymbol{x}=\left( x_{1},\ldots,x_{n} \right)$ is defined as the coefficient of $\boldsymbol{v}=\left( v_{1},\ldots,v_{n} \right)$ in the Taylor series expansion of the cumulant generating function:

$$K\left( \boldsymbol{v} \right)=lnM\left( v \right)E\left\{ e^{j\left\langle v,x \right\rangle} \right\}$$

To compare, the moment generating function is the following:

$$M\left( \boldsymbol{v} \right)=E\left\{ e^{j\left\langle\boldsymbol{v},\boldsymbol{x} \right\rangle} \right\}$$

Therefore, the nth-order cumulants can be inferred from its joint moments of orders up to n as follows:

$$C_{x}\left( I \right)=\sum_{U_{p=1}^{q}I_{p}=I} \left( -1 \right)^{q-1}\left( q-1 \right)!\prod_{p=1}^{q} m_{x}\left( I_{p} \right)$$

Where $U_{p=1}^{q}I_{p}=I$ denotes summation over all partitions of set *I*. See *Table A1 from Mendel et al (1991).*

Property 1: If $\lambda_{i}$, $i=1,\ldots,n,$ are constants, and $x_{i}$, $i=1,\ldots,n,$ are random variables, then

$$\kappa_{n}\left( \lambda_{1}x_{1},\ldots,\lambda_{k}x_{n} \right)=\left( \prod_{i=1}^{n} \lambda_{i} \right)\kappa_{n}\left( x_{1},\ldots,x_{n} \right)$$

Property 2: If $\alpha$ is a constant, then

$$\kappa_{n}\left( {\alpha+x}_{1},\ldots,x_{n} \right)=\kappa_{n}\left( x_{1},\ldots,x_{n} \right)$$

Property 3: If the random variable $\left\{ x_{i} \right\}$ are indepedent of the random variables $\left\{ y_{i} \right\}, i=1,\ldots,n$ then

$$\kappa_{n}\left( x_{1}+y_{1},\ldots,x_{n}+y_{n} \right)\boldsymbol{=}\kappa_{n}\left( x_{1},\ldots,x_{n} \right)+\kappa_{n}\left( y_{1},\ldots,y_{n} \right)$$

As explained previsouly, cross-terms (cross-talks between many fluorophores) are present in moments and absent in cumulants. This difference is formulated by property 3 which is exclusive to cumulants (can be easily demonstrated using « Definition »).

Returning to the equation of $I\left( \vec{r},t \right)$, the intensity distribution measured over time on a detector pixel $\vec{r}$ of a sample composed of M independetly fluctuating emitters can be written as:

$$I\left( \vec{r},t \right)=\sum_{k=1}^{M} \epsilon_{k}U\left( \vec{r}-\vec{r}_{k} \right)s_{k}\left( t \right)+b\left( \vec{r} \right)$$

The n^th^ order cumulants is described by:

$$\kappa_{n}\left\{ I\left( \vec{r},t \right) \right\}\left( \boldsymbol{\tau} \right)=\kappa_{n}\left\{ \sum_{k=1}^{M} \epsilon_{k}U\left( \vec{r}-\vec{r}_{k} \right)s_{k}\left( t \right)+b\left( \vec{r} \right) \right\}\left( \boldsymbol{\tau} \right)\underset{\Rightarrow}{Prop. 2}\kappa_{n}\left\{ \sum_{k=1}^{M} \epsilon_{k}U\left( \vec{r}-\vec{r}_{k} \right)s_{k}\left( t \right) \right\}\left( \boldsymbol{\tau} \right)$$

$$\underset{\Rightarrow}{Prop. 3}\sum_{k=1}^{M} \kappa_{n}\left\{ \epsilon_{k}U\left( \vec{r}-\vec{r}_{k} \right)s_{k}\left( t \right) \right\}\left( \boldsymbol{\tau} \right)\underset{\Rightarrow}{Prop. 1}\sum_{k=1}^{M} \epsilon_{k}^{n}U^{n}\left( \vec{r}-\vec{r}_{k} \right)\kappa_{n}\left\{ s_{k}\left( t \right) \right\}\left( \boldsymbol{\tau} \right)$$

$\epsilon_{k}^{n}U^{n}\left( \vec{r}-\vec{r}_{k} \right)$ contains information about the brightness $\epsilon_{k}^{n}$, location $\vec{r}_{k}$and induced point-spread function $U^{n}\left( \vec{r} \right)$ of the fluorophore $k$. It is straightforward to notice that the position of the fluorophore, as described in $I\left( \vec{r},t \right)$, is unchanged and thus $\kappa_{n}\left\{ I\left( \vec{r},t \right) \right\}$ does not affect the true location of fluorophores. We can also notice that the point-spread function $U\left( \vec{r}-\vec{r}_{k} \right)$ has been replaced by $U^{n}\left( \vec{r}-\vec{r}_{k} \right)$. If we approximate the point-spread function $U\left( \vec{r} \right)$ by a Gaussian function, we obtain:

$$U\left( \vec{r} \right)=e^{-\frac{r^{2}}{2\sigma^{2}}} and U^{n}\left( \vec{r} \right)=e^{-\frac{r^{2}}{2\left( \frac{\sigma}{\sqrt{n}} \right)^{2}}}$$

In that case, the point-spread function of $\kappa_{n}\left\{ I\left( \vec{r},t \right) \right\}$ becomes thinner by a factor $\sqrt{n}$ and as consequence, there is a resolution improvement of a factor $\sqrt{n}$ in $\kappa_{n}\left\{ I\left( \vec{r},t \right) \right\}$ as compared to $I\left( \vec{r},t \right)$. In addition, due to Property 2, the background was in theory removed. Finally, since $\kappa_{n}\left\{ s_{k}\left( t \right) \right\}\left( \boldsymbol{\tau} \right)$ is neither necessary for resolution improvement or background suppression, we arbitrarily compute $\kappa_{n}\left\{ I\left( \vec{r},t \right) \right\}\left( \boldsymbol{0} \right)$ for an impressively fast computation (3-4 seconds for $n=\left\{ 1,2,3,4,5,6,7 \right\}$). Finally, since cumulants are by nature blind to random Gaussian processes, a wide range of time-varying noise are significantly reduced in the super-resolved image as compared to the original diffraction-limited image. In other words, if {x(t)} is Gaussian then the cumulants of {x(t)} are all zero.

The cumulants $\kappa_{n}\left\{ I\left( \vec{r},t \right) \right\}(\boldsymbol{0})$ are presently computed for each pixel detector individually to provide a background-free and noise-reduced super-resolved image. Nevertheless, it is also possible to combine the information of many pixels to even further reduce any time-varying noise and determine inter-pixels values by computing ***cross-cumulants*** ${X\kappa}_{n}\left\{ I\left( \vec{r}_{1},t \right),\ldots,I\left( \vec{r}_{n},t \right) \right\}(\boldsymbol{0})$. In this case, the inter-pixel positions $\vec{r}$ are defined by $\vec{r}=\frac{\vec{r}_{1}+\ldots+\vec{r}_{n}}{n}$.

In the case of 2^nd^ order statistics, the cross-cumulants can be written as

$${X\kappa}_{2}\left\{ I\left( \vec{r}_{1},t \right),I\left( \vec{r}_{2},t \right) \right\}\left( \tau\right)=E\left\{ I\left( \vec{r_{1}},t \right)I\left( \vec{r_{2}},t+\tau\right) \right\}=U\left( \frac{\vec{r}_{1}-\vec{r}_{2}}{\sqrt{2}} \right)\sum_{k=1}^{M} \epsilon_{k}^{2}U^{2}\left( \frac{\vec{r}_{1}+\vec{r}_{2}}{2}-\vec{r}_{k} \right)\left\langle s_{k}\left( t \right),s_{k}\left( t+\tau\right) \right\rangle_{t}$$

To compare: $\kappa_{2}\left\{ I\left( \vec{r_{1}},t \right) \right\}\left( \tau\right)=E\left\{ I\left( \vec{r_{1}},t \right)I\left( \vec{r_{1}},t+\tau\right) \right\}=\sum_{k=1}^{M} \epsilon_{k}^{2}U^{2}\left( \vec{r_{1}}-\vec{r}_{k} \right)\left\langle s_{k}\left( t \right),s_{k}\left( t+\tau\right) \right\rangle_{t}$

assuming a Gaussian point-spread function. As shown in the equation above, ${X\kappa}_{2}$ can be easily computed since it equals the temporal cross-correlation of $I\left( \vec{r_{1}},t \right)$ with $I\left( \vec{r_{2}},t \right)$. Since time-varying noise, including shot noise, is spatially and temporally uncorrelated (noise varies randomly with time and position), the temporal cross-correlation function of noise tends to zero. In addition, the thinner point spread function $U^{2}$ is now centered at $\vec{r}=\frac{\vec{r}_{1}+\vec{r}_{2}}{2}$ instead of $\vec{r_{1}}$, and thus a virtual pixel $\vec{r}$ between $\vec{r}_{1}$ and $\vec{r}_{2}$is computed.

We can further push the reasoning as such: the pixel at location $\vec{r}=\frac{\vec{r}_{1}+\vec{r}_{2}}{2}$ can also be computed with two other neighbouring pixels (i.e. in vertical direction rather than horizontal) $\vec{r}=\frac{\vec{r}_{3}+\vec{r}_{4}}{2}$. We could therefore calculate the pixel value of $\vec{r}$ in two different ways to then average them leading to a reduced amount of noise in the new pixel value. We can extend the reasoning to n^th^ order cross-cumulants where we would need *n* pixels for the computation and thus have a much higher number of ways of computing the cumulant pixel andaveraging some together as described in Dertinger et al. 2009 to even further reduce noise. .

The resulting n^th^ order cross-cumulant can thus be described by the following equation:

$${X\kappa}_{n}\left\{ I\left( \vec{r},t \right) \right\}=\prod_{j<l}^{n} U\left( \frac{r_{j}{-r}_{l}}{\sqrt{n}} \right)\sum_{i=1}^{N} {\epsilon_{k}^{n}U}^{n}\left( r_{i}-\frac{\sum_{k}^{n} r_{k}}{n} \right)\kappa_{n}\left\{ s_{k}\left( t \right) \right\}$$

Figure 1 shows the comparison of ${X\kappa}_{2}\left\{ I\left( \vec{r}_{1},t \right),I\left( \vec{r}_{2},t \right) \right\}\left( \boldsymbol{0} \right)$ with a spline interpolation of $I\left( \vec{r},t_{0} \right)$. Fluorophores are seen “sharper” (due to a thinner point spread function) on ${X\kappa}_{2}\left\{ I\left( \vec{r}_{1},t \right),I\left( \vec{r}_{2},t \right) \right\}\left( \boldsymbol{0} \right)=E\left\{ I\left( \vec{r},t \right)I\left( \vec{r},t+\tau\right) \right\}(\tau=0)$ than on $I\left( \vec{r},{t=t}_{0} \right)$. In addition, in ${X\kappa}_{2}$, noise has been completely removed from $I\left( \vec{r},t \right)$. Figure 2 and 3 helps understanding why only considering ${X\kappa}_{2}\left\{ I\left( \vec{r}_{1},t \right),I\left( \vec{r}_{2},t \right) \right\}\left( \boldsymbol{0} \right)$ and not ${X\kappa}_{2}\left\{ I\left( \vec{r}_{1},t \right),I\left( \vec{r}_{2},t \right) \right\}\left( \tau\right), \tau\in\mathbb{R}^{+}$ or $\int{X\kappa}_{2}\left\{ I\left( \vec{r}_{1},t \right),I\left( \vec{r}_{2},t \right) \right\}\left( \tau\right)d\tau$ is appropriate. Indeed, since there are no practical gain in reducing noise and improving the resolution, computing only ${X\kappa}_{2}(0)$ is sufficient. Only the first few coefficients $\left( \tau\sim0 \right)$ provide sufficient information: ${X\kappa}_{2}\left( \tau\right)$ rapidly decays toward zero as $\tau$ increases.
