# Supplementary material for: SOFI Simulation Tool: A Software Package for Simulating and Testing Super-Resolution Optical Fluctuation Imaging
Source: PLoS One. 2016 Sep 1;11(9):e0161602. doi: 10.1371/journal.pone.0161602 (PMC5008722; doi:10.1371/journal.pone.0161602)
Supplement: S2 Appendix — Zip file which includes the software package. The software is written in MATLAB, equipped with graphical user interface and freely available together with a user manual also at [16]. (ZIP) [file pone.0161602.s002.zip › sofisimulationtool-2016-07-12/GUI/codeUtils/help_TextFigures/axes7/axes7.docx]

**Flattening**

The n^th^ order cross-cumulant can be described by the following equation:

$${X\kappa}_{n}\left\{ I\left( \vec{r},t \right) \right\}=\prod_{j<l}^{n} U\left( \frac{r_{j}{-r}_{l}}{\sqrt{n}} \right)\sum_{i=1}^{N} {\epsilon_{k}^{n}U}^{n}\left( r_{i}-\frac{\sum_{k}^{n} r_{k}}{n} \right)\kappa_{n}\left\{ s_{k}\left( t \right) \right\}$$

This equation describes the nth order cross-cumulant computed and shown in the previous step (Raw Cumulants). The sum in equation (1) is weighted by the product of *n* PSF-shaped weight factors, called the ***distance factor****,* which depends on the distances ***d_jl_*** between **r**_j_ and **r**_l_, *j* and *l* spanning the pixel set used for cross-cumulant computation (see Raw Cumulants). As ***d*** increases, r_j_ and r_l_ become further apart reducing the value of the distance up to a certain point when the cross-cumulant ${X\kappa}_{n}$ approaches zero. As a consequence, only nearby pixels (i.e. a small distance ***d*** between **r**_j_ and **r**_l_) can be used to compute cross cumulants so as to reduce as much as possible the error introduced by this weighting/distance factor. However, as the order of SOFI grows, so does the error introduced by the weighting factor.

Indeed, Figure 1 depicts a 4^th^ order 1D SOFI grid where light gray squares with single letters correspond to the physical pixels of the camera and light blue squares with four letters to the inter-pixels computed with cross-cumulants. The error introduced in this case by the distance factor can be expressed in the following terms: the inter-pixel exactly between physical pixel A and B (pixel AABB) will have a different weighting factor than the one closer to A (pixel AAAB) since the distance between them and the physical pixels is different. Therefore, both inter-pixels (AAAB and AABB) have distinct weighting factor.

An elegant way to resolve this issue is to multiply each cross-cumulant pixel by the inverse of its corresponding distance factor (which we can compute by estimating the PSF $U\left( \boldsymbol{r} \right)$ of the optical system). The cross-cumulants after flattening become:

$${X\kappa}_{n}\left\{ I\left( \vec{r},t \right) \right\}\approx\sum_{i=1}^{N} {\epsilon_{k}^{n}U}^{n}\left( r_{i}-\frac{\sum_{k}^{n} r_{k}}{n} \right)\kappa_{n}\left\{ s_{k}\left( t \right) \right\}$$

Nevertheless, the estimation of$U\left( \boldsymbol{r} \right)$ can quite often be cumbersome and based on large approximations. Therefore we have developed a simple and robust method in order to correct for the weighting factor which gives either a better or similar result as compared to estimating the inverse of the distance factor.

Figure 2.a. depicts a 2^nd^ order 3x3 SOFI grid where light gray squares with single letters again correspond to the physical pixels on the camera and light blue squares with two letters to the inter-pixels (or virtual pixels) computed with cross-cumulants. This grid can be decomposed into two separate grids, a grid containing all the physical pixels and another containing all the inter-pixels, shown in Figure 2.b and 2.c respectively. Both grids describe the same confined region in the sample and should hence share the same variance. Therefore, by multiplying the inter-pixel grid (Figure 2.c.) with the ratio between the variance of the physical pixels and the variance of the virtual pixels, the inter-pixels values are brought in the same range of the physical pixel values removing the weighting factor’s effect.
