# Supplementary material for: SOFI Simulation Tool: A Software Package for Simulating and Testing Super-Resolution Optical Fluctuation Imaging
Source: PLoS One. 2016 Sep 1;11(9):e0161602. doi: 10.1371/journal.pone.0161602 (PMC5008722; doi:10.1371/journal.pone.0161602)
Supplement: S2 Appendix — Zip file which includes the software package. The software is written in MATLAB, equipped with graphical user interface and freely available together with a user manual also at [16]. (ZIP) [file pone.0161602.s002.zip › sofisimulationtool-2016-07-12/GUI/codeUtils/help_TextFigures/axes8/axes8.docx]

**Linearization**

At this point, noise and background were either eliminated or reduced, and the resolution was improved by$\sqrt{n}$. It is nevertheless very important to notice that the brightness $\epsilon_{k}$ of each fluorophore is greatly affected in the process of cross-cumulant computation which, at high orders, alters dramatically the contrast of the SOFI image: small differences in brightness between fluorophores are severely amplified. For example, an emitter that has a 2-fold larger molecular brightness $\epsilon_{k}$than another will appear $2^{n}$ times brighter than the other in the n^th^ order SOFI image. In addition, an emitter that does not fluctuate over time will yield a null cumulant and will thus not appear in the SOFI image. These issues will thereafter be respectively referred to as **amplified brightness** and **blinking heterogeneities**.

The flattened n^th^ order cross-cumulant can be described by the following equation:

$${X\kappa}_{n}\left\{ I\left( \vec{r},t \right) \right\}=\sum_{i=1}^{N} {\epsilon_{k}^{n}U}^{n}\left( r_{i}-\frac{\sum_{k}^{n} r_{k}}{n} \right)\kappa_{n}\left\{ s_{k}\left( t \right) \right\}$$

where $s_{k}\left( t \right)$ is a time-varying stochastic signal that equals 1 when the emitter is in the on-state and $\xi\in[0,1[$ when it is in the off-state. The relative durations of the on and off-states are respectively:

$$\rho_{on}=\frac{\tau_{on}}{\tau_{on}+\tau_{off}}, and \rho_{off}=\frac{\tau_{off}}{\tau_{on}+\tau_{off}}=1-\rho_{on}$$

This blinking behaviour $s_{k}\left( t \right)$ can thus be described by:

$$s_{k}\left( t \right)={\left( 1-\xi_{k} \right)f}_{k}\left( t \right)$$

$f_{k}\left( t \right)$ defined by a Bernouilli distribution with probability $\rho_{on}$. In other words, the fluorophore is either in the on state with probability $\rho_{on}$ or in the off-state with probability $1-\rho_{on}$. Therefore, the cumulant of the stochastic signal $s_{k}\left( t \right)$ is:

$$\kappa_{n}\left\{ s_{k}\left( t \right) \right\}=\kappa_{n}\left\{ {\left( 1-\xi_{k} \right)f}_{k}\left( t \right) \right\}={{f_{n}\left( \rho_{on,k} \right)=\left( 1-\xi_{k} \right)}^{n}\rho}_{on,k}\left( 1-\rho_{on,k} \right)\frac{\partial\kappa_{n-1}\left\{ s_{k}\left( t \right) \right\}}{\partial\rho_{on,k}}$$

With $\kappa_{1}\left\{ s_{k}\left( t \right) \right\}={\left( 1-\xi_{k} \right)^{1}\rho}_{on,k}$ and ${\kappa_{2}\left\{ s_{k}\left( t \right) \right\}=\left( 1-\xi_{k} \right)^{2}\rho}_{on,k}\left( 1-\rho_{on,k} \right)$

Combining this result with equation (1) leads to the following n^th^ order cross-cumulant:

$${X\kappa}_{n}\left\{ I\left( \boldsymbol{r}_{\boldsymbol{S}},t \right) \right\}=\sum_{i=1}^{N} {\epsilon_{k}^{n}U}^{n}\left( \boldsymbol{r}_{i}-\frac{\sum_{k}^{n} \boldsymbol{r}_{k}}{n} \right)f_{n}\left( \rho_{on,k} \right)={\epsilon^{n}\left( \boldsymbol{r} \right)f}_{n}\left( \rho_{on};\boldsymbol{r} \right)\sum_{i=1}^{N} U^{n}\left( \boldsymbol{r}_{i}-\frac{\sum_{k}^{n} \boldsymbol{r}_{k}}{n} \right)$$

where both $\epsilon^{n}$ and $f_{n}$ were written to vary according to spatial coordinates instead of individual fluorophores (i.e. $\epsilon\left( \vec{r} \right)\approx\sum\epsilon_{k}$).

In order to correct for the amplified brightness $\epsilon_{k}^{n}$, the cumulants are deconvolved with an estimate of the point-spread function. Assuming a perfect deconvolution, the result can be interpreted as follows:

$${X\kappa}_{n}\left\{ I\left( \boldsymbol{r}_{1},t \right)\ldots I\left( \boldsymbol{r}_{n},t \right) \right\}={\epsilon^{n}\left( \boldsymbol{r} \right)f}_{n}\left( \rho_{on};\boldsymbol{r} \right)\sum_{i=1}^{N} \delta\left( r_{i}-\frac{\sum_{k}^{n} r_{k}}{n} \right)$$

Since each point-spread function has been reduced to a Dirac pulse, taking the n^th^ root linearizes the brightness response without cancelling the resolution improvement of the cumulant. The linearized cumulant can be expressed by:

$${X\kappa}_{n}\left\{ I\left( \boldsymbol{r},t \right) \right\}={\epsilon\left( \boldsymbol{r} \right)\left| f_{n} \right|}^{\frac{1}{n}}\left( \rho_{on};\boldsymbol{r} \right)\sum_{i=1}^{N} \delta\left( \boldsymbol{r}_{i}-\frac{\sum_{k}^{n} \boldsymbol{r}_{k}}{n} \right)$$
