# Supplementary material for: SOFI Simulation Tool: A Software Package for Simulating and Testing Super-Resolution Optical Fluctuation Imaging
Source: PLoS One. 2016 Sep 1;11(9):e0161602. doi: 10.1371/journal.pone.0161602 (PMC5008722; doi:10.1371/journal.pone.0161602)
Supplement: S2 Appendix — Zip file which includes the software package. The software is written in MATLAB, equipped with graphical user interface and freely available together with a user manual also at [16]. (ZIP) [file pone.0161602.s002.zip › sofisimulationtool-2016-07-12/GUI/codeUtils/help_TextFigures/axes9/axes9.docx]

**Reconvolution**

The linearized cross-cumulant is defined by the following equation:

$${X\kappa}_{n}\left\{ I\left( \boldsymbol{r},t \right) \right\}={\epsilon\left( \boldsymbol{r} \right)\left| f_{n} \right|}^{\frac{1}{n}}\left( \rho_{on};\boldsymbol{r} \right)\sum_{i=1}^{N} \delta\left( \boldsymbol{r}_{i}-\frac{\sum_{k}^{n} \boldsymbol{r}_{k}}{n} \right)$$

In order to mask artifacts introduced by deconvolution (see Linearization), small values are truncated and the image is reconvolved with a scaled version of the PSF $U\left( \vec{r} \right)$of the optical system.

The scale of the point-spread function used for reconvolution is chosen so that the scaled OTF has the same frequency support than the n^th^ order cross-cumulant equivalent optical transfer function. In fact, the Fourier transform of a product of n functions equals the convolution between the Fourier transform of each function. In addition, since the support of the convolution of an OTF with itself has twice its original support, the support of the cumulant OTF has n times the support of the system’s OTF, and therefore, the image is reconvolved by $U\left( n\vec{r} \right)$:

$$U^{n}\left( \vec{r} \right)\underset{\to}{\mathfrak{I}ourier}O\left( \vec{k} \right)=\hat{U}\left( \vec{k} \right)\underset{(n-1)times}{\underbrace{*\ldots*}}\hat{U}\left( \vec{k} \right) \underset{\to}{Deconv. and Reconv}O'\left( \vec{k} \right)=\hat{U}\left( n\vec{k} \right)\underset{\to}{{\mathfrak{I}ourier}^{-1}}U\left( \frac{\vec{r}}{n} \right)$$

Assuming a Gaussian point-spread function, the resolution is improved by a factor *n* (instead of $\sqrt{n}$ as it was initially).

Before linearization, the cross-cumulant was described by:

$${X\kappa}_{n}\left\{ I\left( \vec{r},t \right) \right\}=\sum_{i=1}^{N} {\epsilon_{k}^{n}U}^{n}\left( r_{i}-\frac{\sum_{k}^{n} r_{k}}{n} \right)\kappa_{n}\left\{ s_{k}\left( t \right) \right\}$$

After linearization and convolution, the cross-cumulant is now expressed by:

$${X\kappa}_{n}\left\{ I\left( \boldsymbol{r},t \right) \right\}={\epsilon\left( \boldsymbol{r} \right)\left| f_{n} \right|}^{\frac{1}{n}}\left( \rho_{on};\boldsymbol{r} \right)\sum_{i=1}^{N} U\left( \frac{\boldsymbol{r}_{i}-\frac{\sum_{k}^{n} \boldsymbol{r}_{k}}{n}}{n} \right)$$

Resulting in an improved resolution (by a factor $\sqrt{n}$ between the two) and a linearized brightness $\epsilon$ resolving the amplified brightness issue described in previous tutorial steps.
